# Localized therapeutic strategy based on microRNA-21-loaded mesoporous silica nanoparticles hydrogel improves bone repair in medication-related osteonecrosis of the jaw

**DOI:** 10.1186/s13018-025-06503-7

**Published:** 2025-12-23

**Authors:** Ye Li, Siyuan Huang, Yihan Xing, Zhuofan Chen, Dongsheng Yu

**Affiliations:** 1 Hospital of Stomatology, Guanghua School of Stomatology, Institute of Stomatological Research, Sun Yat-Sen University, Guangzhou, 510000 Guangdong China; 2 Guangdong Provincial Key Laboratory of Stomatology, Sun Yat-Sen University, Guangzhou, 510000 Guangdong China

**Keywords:** Medication-related osteonecrosis of the jaw, miR-21, Mesoporous silica nanoparticles, Hydrogel drug delivery system, Osteoclast, Osteoblast

## Abstract

**Background:**

Medication-related osteonecrosis of the jaw (MRONJ), caused by long-term anti-resorptive therapy, leads to bone necrosis and impaired healing. This study developed a miR-21-loaded mesoporous silica nanoparticles (MSN) hydrogel to promote local bone regeneration.

**Methods:**

Transcriptome sequencing of zoledronic acid (ZOL)-treated osteoclasts (OCs) revealed PDCD4 upregulation and NF-κB (p65) phosphorylation inhibition. miR-21, identified as a regulator of PDCD4, was validated in vitro. Amino-modified mesoporous silica nanoparticles (MSN-NH₂) were synthesized to carry miR-21, and embedded in a dynamic Schiff base-crosslinked hydrogel. The hydrogel's biocompatibility, sustained release, and therapeutic effect were evaluated in a rat MRONJ model via micro-CT, histology, TRAP staining, and RNA in situ hybridization (RISH).

**Results:**

miR-21 reversed ZOL-induced suppression of NF-κB/p65 phosphorylation by targeting PDCD4, thereby restoring osteoclast differentiation and resorption activity. The miR-21-loaded MSN hydrogel promoted bone regeneration, increased TRAP⁺ osteoclast numbers, and elevated local miR-21 expression, while maintaining systemic safety. These findings suggest that the miR-21-loaded MSN hydrogel system exerts its therapeutic effect primarily through the miR-21/PDCD4/NF-κB signaling pathway, facilitating coordinated regulation of osteoclast-mediated bone remodeling.

**Conclusions:**

The miR-21-loaded MSN hydrogel effectively restored bone remodeling and healed MRONJ defects without systemic toxicity, offering a promising localized adjunct to anti-resorptive therapies.

**Supplementary Information:**

The online version contains supplementary material available at 10.1186/s13018-025-06503-7.

## Introduction

Medication-related osteonecrosis of the jaw (MRONJ) is an uncommon yet severe consequence of antiresorptive and antiangiogenic treatments, resulting in ongoing bone deterioration in the maxillofacial area [1]. Patients may exhibit various oral issues that adversely affect their quality of life [2]. The initial instances of jaw osteonecrosis were identified in 2003 in connection with two bisphosphonates, namely pamidronate and zoledronate [3]. Subsequently, other drugs, including denosumab and those with antiangiogenic properties—such as tyrosine-kinase inhibitors (TKIs), vascular endothelial growth factor (VEGF) inhibitors, and the mammalian target of rapamycin (mTOR)—have also been linked to the development of osteonecrosis [4–7]. Notably, antiresorptive agents are widely used for osteoporosis as well as cancer-related bone disease, and excessive suppression of jawbone remodeling has been implicated as a key mechanism underlying MRONJ in susceptible patients [8, 9]. While these medications can provide considerable benefits in treating the underlying disease, it is crucial to focus on the prevention and management of MRONJ.

MicroRNAs (miRNAs) are a class of small non-coding ribonucleic acids. Emerging studies have emphasized that miRNAs are promising therapeutic candidates, with more than 2,000 human miRNAs involved in regulating various disease processes [10], such as tendon injuries [11, 12], osteoarthritis [13], rheumatoid arthritis [14], and osteoporosis [15–17]. Among them, specific miRNAs involved in osteoclast differentiation and bone remodeling have shown particular relevance to MRONJ, where impaired osteoclast function is central to its pathogenesis [18]. miR-21 has been extensively studied in bone biology and has been shown to have an important regulatory role in both osteogenesis and osteoblastogenesis [19]. miR-21 influences the regulation of Suppressor of Mothers against Decapentaplegic7 (Smad7), sprouty RTK signaling antagonist 1 (Spry1), and periodontal-ligament-associated protein-1 (PLAP1) to promote osteogenic differentiation [20–22]. Additionally, in MRONJ, miR-21 has been reported to promote osteoclastogenesis by upregulating receptor activator of nuclear factor kappa-B ligand (RANKL) and downregulating osteoprotegerin, thereby disturbing bone remodeling homeostasis [19, 23]. This highlights the therapeutic potential of miR-21 in restoring MRONJ bone homeostasis. However, the clinical application of miRNA-based therapy remains limited due to rapid enzymatic degradation, off-target effects, and inefficient tissue targeting [24, 25]. To address these challenges, mesoporous silica nanoparticles (MSN) have emerged as promising delivery platforms owing to their excellent biocompatibility, large surface area, tunable pore size, and intrinsic osteoinductive capacity [26, 27]. When incorporated into hydrophilic polymer–based hydrogels, MSNs can provide spatiotemporally controlled release of miRNAs, thereby enhancing local stability and bioactivity while minimizing systemic exposure [28–30].

Here, we aimed to develop a miR-21-based delivery system to improve local bone healing in MRONJ, offering both novelty and translational potential. Transcriptomic sequencing was first used to identify differentially expressed miRNAs associated with osteoclast dysfunction under zoledronic acid (ZOL) treatment, revealing that miR-21 may restore osteoclast function by targeting PDCD4 and modulating the NF-κB pathway. Based on this, a miR-21 delivery system was constructed using amino-modified mesoporous silica nanoparticles (MSN-NH_2_), which were further incorporated into a preformed hydrogel crosslinked with oxidized hyaluronic acid and gelatin for sustained local release. A series of in vitro and in vivo experiments confirmed the system's efficacy in enhancing osteoclast activity, promoting bone regeneration, and repairing mandibular defects in MRONJ. This study provides a novel miRNA-based local therapeutic strategy for MRONJ and broadens the application prospects of miRNA delivery systems in bone regeneration.

## Materials and methods

### Target miRNA prediction

In this study, we performed miRNA prediction analysis for PDCD4 mRNA using the StarBase v2.0 (http://starbase.sysu.edu.cn/). StarBase provides comprehensive predictions of miRNA-target gene interactions and supports data queries across multiple species [31]. Specifically, we input PDCD4 mRNA as the target gene and used the miRNA prediction tools provided by the database to identify potential miRNAs that bind to PDCD4.

### Cell acquisition and culture

Mouse mononuclear macrophages (RAW 264.7) and pre-osteoblastic cells (MC3T3-E1) cell lines were purchased from the Cell Bank of the Chinese Academy of Sciences (Shanghai, China) and authenticated by short tandem repeat (STR) profiling. RAW 264.7 cells were cultured in Dulbecco’s Modified Eagle Medium (DMEM; Gibco, Australia) supplemented with 10% fetal bovine serum (FBS; Wisent, Canada) and 1% penicillin–streptomycin (Gibco, Australia), and maintained in a humidified incubator with 5% CO_2_ at 37 °C. MC3T3-E1 cells were cultured in α-Minimum Essential Medium (α-MEM) supplemented with 10% FBS and 1% penicillin–streptomycin under the same incubation conditions. Both cell lines were passaged when reaching 70–80% confluence using gentle pipetting.

Bone marrow mononuclear cells (BMMNCs) were isolated from the femoral bone marrow of 6-week-old Sprague–Dawley (SD) rats as previously described [32]. BMMNCs were seeded into 12-well plates at a density of 5 × 10^4^ cells/well and cultured in the presence of M-CSF (10 ng/mL) and RANKL (75 ng/mL; Novoprotein, China). Cells were randomly divided into two groups: Control (CTR) group and ZOL treatment group (1 μM ZOL; MedChemExpress, China). After 72 h of induction, total RNA was extracted using TRIzol reagent (Thermo Fisher Scientific, USA). RNA sequencing and data processing were performed by the BGI Genomics Institute (Shenzhen, China), and differentially expressed genes were identified through the Dr. Tom analysis platform (http://report.bgi.com).

### Cell transfection

RAW 264.7 cells were seeded in 6-well plates at a density of 2 × 10^4^ cells per well and incubated overnight. Cells were then randomly divided into four groups for transfection: miR-21 mimics group, miR-21 inhibitor group, mimics negative control (m-NC) group, and inhibitor negative control (i-NC) group. Transfections were carried out using a miR-21 mimic or inhibitor and corresponding negative control oligonucleotides (RiboBio, China), following the manufacturer's instructions, with the aid of Advanced DNA/RNA Transfection Reagent (RiboBio, China). After a 10-min incubation for complex formation at room temperature, the culture medium was replaced with fresh DMEM (Gibco, Australia) containing 10% fetal bovine serum (FBS; Wisent, Canada) and 1% penicillin–streptomycin. Cells were further incubated for 24 h at 37 °C in a 5% CO_2_ humidified atmosphere. Following transfection, cells were collected and subjected to downstream analyses.

### Cell viability

RAW 264.7 and MC3T3-E1 cells were seeded into 96-well plates at a density of 1,000 cells per well. After 48 h of incubation, 10 μL of CCK-8 reagent (Yeasen, China) was added to each well and incubated for an additional 2 h at 37 °C. The absorbance at 450 nm was then measured using a microplate reader (SkanIt RE 7.0, Thermo Fisher Scientific) [33].

### Tartrate resistant acid phosphatase (TRAP) staining assay

RAW 264.7 cells were seeded into 96-well plates and incubated overnight. The following day, cells were treated with RANKL (75 ng/mL) and/or zoledronic acid (ZOL, 1 μM) for 72 h to induce osteoclast differentiation. After treatment, cells were stained using a TRAP Staining Kit (Nanjing Jiancheng, China) according to the manufacturer's instructions [34]. Multinucleated cells (≥ 3 nuclei) with positive TRAP staining were identified as mature osteoclasts (OCs) under an optical microscope (Olympus IX83+DP74, Japan). The number of TRAP-positive OCs was counted in five randomly selected fields per well (each field covering 2800 mm^2^), and the average number per field was calculated for statistical analysis.

### Bone resorption assay

RAW 264.7 cells were seeded into Osteo Assay 24-well plates (Corning, USA) at a density of 12,000 cells per well and treated with RANKL (75 ng/mL) with or without ZOL (1 μM) for 72 h. After treatment, wells were washed with PBS and stained with 1% Toluidine Blue solution (Solarbio, China). Bone resorption pits were observed under an optical microscope, and five visual fields (615 mm^2^ per field) were captured for each group [35]. The resorption area was quantified using ImageJ software (v1.53, NIH, USA).

### Alkaline phosphatase (ALP) staining assay

MC3T3-E1 cells were seeded into 24-well plates and divided into four groups based on treatment conditions: a Blank group cultured in standard medium without any treatment, an OIM group treated with osteogenic induction medium (OIM), an OIM+MSN group treated with both OIM and mesoporous silica nanoparticles (MSN), and an MSN group treated with MSN alone in standard medium. The OIM consisted of α-MEM supplemented with 10% FBS, 1% penicillin–streptomycin, 50 μg/mL ascorbic acid, 10 mM β-glycerophosphate, and 100 nM dexamethasone. Cells were cultured in 24-well plates with 500 μL medium per well, and the induction medium was refreshed every 2–3 days. After 7 days of incubation, cellular osteogenic differentiation was assessed using an ALP Staining Kit (Beyotime, China) according to the manufacturer’s protocol [36].

### Reverse transcription quantitative PCR (RT-qPCR)

RAW 264.7 and MC3T3-E1 cells were seeded into 24-well plates and cultured under respective experimental conditions. Total RNA was extracted using RNAzol reagent (MRC, USA) according to the manufacturer’s protocol. Reverse transcription was performed using the HiScript III qRT SuperMix (Vazyme, China) to synthesize complementary DNA (cDNA). Gene-specific primers (listed in Table 1) were designed and synthesized by Generay Biotechnology (China). Quantitative PCR was conducted using Taq Pro Universal SYBR qPCR Master Mix (Vazyme, China) on a LightCycler 480 system (Roche, USA). Relative mRNA expression levels were calculated using the 2^−ΔΔCt^ method and normalized to GAPDH as the internal control [37].

**Table 1:** Primers for RT-qPCR

Gene	Primer
Rela (p65)	Forward 5’- ATGGCAGACGATGATCCCTAC -3’ Reverse 5’- CGGAATCGAAATCCCCTCTGTT -3’
Acp5 (TRAP)	Forward 5’- CACTCCCACCCTGAGATTTGT -3’ Reverse 5’- CCCCAGAGACATGATGAAGTCA -3’
Pdcd4	Forward 5’- ACTGACCCTGACAATTTAAGCG -3’ Reverse 5’- TTTTCCGCAGTCGTCTTTTGG -3’
Cathepsin K (CTSK)	Forward 5’- CTCGGCGTTTAATTTGGGAGA -3’ Reverse 5’- TCGAGAGGGAGGTATTCTGAGT -3’
Nfatc1	Forward 5’- CAGTGTGACCGAAGATACCTGG -3’ Reverse 5’- TCGAGACTTGATAGGGACCCC -3’
Alp	Forward 5’-CCAACTCTTTTGTGCCAGAGA-3’ Reverse 5’-GGCTACATTGGTGTTGAGCTTTT-3’
Runx2	Forward 5’-AGAGTCAGATTACAGATCCCAGG-3’ Reverse 5’-TGGCTCTTCTTACTGAGAGAGG-3’
Col1a1	Forward 5’- GCTCCTCTTAGGGGCCACT-3’ Reverse 5’- ATTGGGGACCCTTAGGCCAT-3’
U6	Forward 5’- TCCGATCGTGAAGCGTTC -3’ Reverse 5’- GTGCAGGGTCCGAGGT -3’
miR-21	Forward 5’- TCCGAAGTTGTAGTCAGACT -3’ Reverse 5’- GTGCAGGGTCCGAGGT ‑3’
Gapdh	Forward 5’- TGGCCTTCCGTGTTCCTAC -3’ Reverse 5’- GAGTTGCTGTTGAAGTCGCA -3’

### Western blotting

RAW 264.7 cells were seeded into 6-well plates at a density of 80,000 cells per well and cultured for 72 h. Total protein was extracted using RIPA lysis buffer (CWBIO, China) and quantified with a BCA Protein Assay Kit (Yeasen, China). Equal amounts of protein (10 μL per sample) were separated by SDS-PAGE (Epizyme, China) and transferred onto PVDF membranes (Millipore, USA) [38]. Membranes were blocked with 5% non-fat milk and incubated overnight at 4 °C with primary antibodies against GAPDH, CTSK, p65, phospho-p65 (Ser536), and PDCD4 (all 1:1000; Affinity, China). After washing with TBST, membranes were incubated with HRP-conjugated secondary antibody (1:5000; Yeasen, China) at room temperature. Protein bands were visualized using Super ECL Detection Reagent (Yeasen, China) and imaged on an X-ray film system (Bio-Rad, USA). Band intensities were quantified using Image Lab software (v4.1; Bio-Rad, USA).

### Preparation of injectable O-HA/gelatin hydrogel loaded with MSN/miR-21 complex

Oxidized hyaluronic acid (O-HA) was synthesized by dissolving 1 g of hyaluronic acid (HA; Macklin, China) in 100 mL of deionized water, followed by the addition of 210 mg of sodium periodate (NaIO₄; Aladdin, China) under dark conditions with stirring for 5 h. Ethylene glycol (1 mL, 98%; Macklin, China) was added to quench excess NaIO_4_ [39]. The reaction mixture was dialyzed (MWCO: 8000–14000 Da) for 48 h and lyophilized to obtain O-HA powder.

Mesoporous silica nanoparticles (MSN-NH_2_) were synthesized using a CTAB-templated method. Briefly, 1.12 g CTAB (Macklin, China) was dissolved in 1 L of deionized water (pH adjusted to 11 with 1 M NaOH), followed by the dropwise addition of 5.8 mL tetraethyl orthosilicate (TEOS; Macklin, China) [40]. After stirring for 2 h and aging overnight at room temperature, the product was washed and hydrothermally treated at 120 °C for 72 h. The obtained powder was functionalized by stirring with 1 mL of APTES (50% in ethanol) in 50 mL of anhydrous ethanol for 8 h. CTAB templates were removed by refluxing in acidic methanol (9 mL HCl/400 mL methanol) at 70 °C for 36 h. The final MSN-NH₂ product was washed and vacuum-dried.

To prepare the preformed hydrogel, MSN-NH₂ was incubated with miR-21 mimics (RiboBio, China) at a mass ratio of 10:1 (MSN: miR-21) at 4 °C for 30 min to form MSN/miR-21 complexes. Subsequently, 150 mg O-HA and 80 mg gelatin (Macklin, China) were dissolved in 1 mL of MSN/miR-21 complex solution (containing 8 mg MSN-NH₂ and 0.08 mg miR-21, pH 7.4) [28]. The mixture was incubated at room temperature for 5 min, allowing in situ hydrogel formation via Schiff base linkage between aldehyde groups of O-HA and amino groups of gelatin. The surface morphology of MSN-NH2 and Gel/MSN+miR-21 system was observed by using a scanning electron microscope (SEM, SU8220, HITACHI, Japan). Its chemical composition was examined via energy-dispersive X-ray spectroscopy (EDS, SU8220, HITACHI, Japan). Finally, O-HA, MSN-NH2 and Gel/MSN+miR-21 system were scanned by FTIR (Nicolet iS 10, Thermo Fisher, USA) from 4000 to 400 cm-1. Spectra analysis was performed using Origin (version 2021, OriginLab Corporation, USA) software.

### Establishment of MRONJ rat model and surgical procedure

All animal procedures were approved by the Institutional Animal Care and Use Committee of Sun Yat-sen University. Twenty female Sprague–Dawley (SD) rats (6–8 weeks old, 176 ± 4 g; Zhuhai BesTest Bio-Tech, China) were housed under standard conditions (22 ± 2 °C, 50 ± 10% humidity, 12-h light/dark cycle) with access to regular chow and water. Rats were randomly assigned to four groups: Control, Blank, MSN, and MSN+miR-21. Except for the Control group (which received saline), all other groups were administered ZOL (ZOL, 0.02 mg/kg, i.v., once weekly; 1 μM in PBS) and dexamethasone (DEX, 5 mg/kg, s.c., three times per week) for 8 weeks.

Following this treatment period, rats were anesthetized with Zoletil 50 (1 mL/kg), and a 1 cm horizontal incision was made along the lower margin of the mandible to expose the alveolar bone near the first and second molars. A standardized bone defect (5 × 2 × 3 mm) was created using a low-speed dental drill under continuous saline irrigation. MSN or MSN+miR-21 hydrogels were applied to the respective defect sites, while the Control and Blank groups received no material. The surgical wound was sutured in layers, and the crown of the first mandibular molar was fractured, leaving the root in place. ZOL and DEX administration continued postoperatively for all non-control groups. Mandibles and femora were harvested 8 weeks after surgery for further analysis.

### Micro-CT analysis

Harvested mandibles and femora were scanned using micro-computed tomography (micro-CT) with consistent scanning parameters [41, 42]. CT data were reconstructed and analyzed using Mimics Research (v23.0, Materialise, Belgium) and CTAn software (v1.23, Bruker, Germany). For the mandibular defect site, a region of interest (ROI) measuring 5 × 3 × 2 mm^3^ was defined within the bone defect area to evaluate new bone microarchitecture. Quantitative parameters, including bone mineral density (BMD, g/cm^3^), bone volume (BV, mm^3^), total volume (TV, mm^3^), and the bone volume fraction (BV/TV, %) were calculated. For femoral bone, BMD, trabecular thickness (Tb.Th, mm), trabecular number (Tb.N, 1/mm), and trabecular separation (Tb.Sp, mm) were also assessed.

### Histological analysis

Mandibles and femora were decalcified in EDTA solution (Servicebio, China) for three weeks, followed by graded ethanol dehydration, paraffin embedding, and sectioning into 5 μm-thick slices using a rotary microtome (Leica Microsystems, Germany). For mandibular specimens, hematoxylin and eosin (H&E) staining [43], tartrate-resistant acid phosphatase (TRAP) staining (Nanjing Jiancheng, China), and microRNA in situ hybridization (RISH) [44] for miR-21 were performed. For femora and major visceral organs (heart, liver, spleen, lung, and kidney), H&E staining was conducted to evaluate systemic toxicity. Histological images were acquired using a polarized light microscope (Nikon, Japan) and analyzed with Slide Viewer software (v2.5, 3DHISTECH, Hungary).

### Statistical analysis

Statistical analyses were conducted using GraphPad Prism (v9.0, GraphPad Software). Data are presented as mean ± standard deviation (M ± SD). One-way or two-way analysis of variance (ANOVA) was used to evaluate differences among groups, followed by Tukey’s or Dunnett’s post hoc tests for multiple comparisons, with a significance level set at α = 0.05. Statistical significance was indicated as follows: *P < 0.05, **P < 0.01, ***P < 0.001, ****P < 0.0001.

## Results

### Preliminary exploration of the mechanism by which ZOL affects NF-κB pathway activation and miR-21/PDCD4 expression in osteoblasts (OBs)

**Fig. 1:**
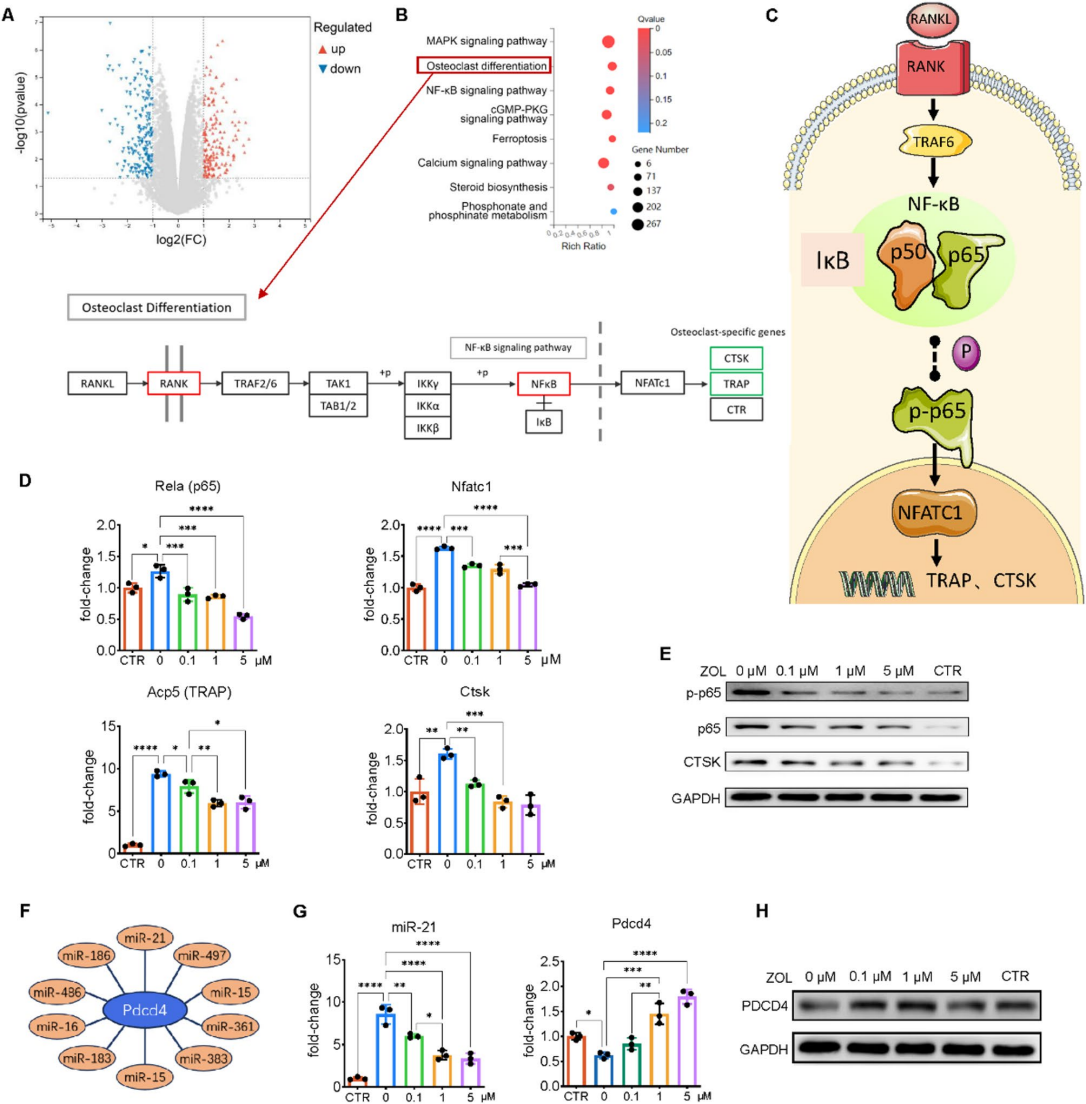
RNA-sequencing analysis of OCs cultured with ZOL. **A** Volcano plot showcasing differentially expressed genes in ZOL and Control groups; **B** KEGG pathway enrichment of differentially expressed genes based on ZOL and Control groups, and the expression of related genes in the entry for osteoclast differentiation; **C** Schematic of osteoclast differentiation pathway; **D** Gene expression across different groups were evaluated using RT-qPCR; **E** Western blot assays measuring the related protein expression; **F** The miRNA-Pdcd4 regulatory network; **G**, **H** Pdcd4 and miR-21 expression across different groups were evaluated using RT-qPCR (**G**) and western blot (**H**). Data are presented as mean ± standard deviation (SD) from three independent experiments. Statistical significance among multiple groups was determined using one-way ANOVA followed by Tukey’s post hoc test. *P < 0.05; **P < 0.01; ***P < 0.001; ****P < 0.0001

To explore the potential mechanisms by which ZOL affects osteoclast function, primary BMMNCs isolated from SD rats were induced to differentiate into OCs using M-CSF and RANKL, followed by ZOL treatment for 3 days. RNA sequencing was performed on ZOL-treated and untreated cells. The volcano plot (Fig. 1A) revealed substantial transcriptional differences between the two groups. The kyoto encyclopedia of genes and genomes (KEGG) enrichment analysis (Fig. 1B) identified “osteoclast differentiation” and “NF-κB signaling pathway” as significantly enriched pathways. This suggests that ZOL intervention may affect the osteoclast differentiation pathway (Fig. 1C). To validate these transcriptomic findings, RAW 264.7 cells were induced to differentiate into OCs and treated with increasing concentrations of ZOL. RT-qPCR (Fig. 1D) confirmed that ZOL significantly reduced the mRNA levels of Rela (p65), Nfatc1, Acp5, and Ctsk in a dose-dependent manner. Correspondingly, Western blotting (Fig. 1E) demonstrated a marked reduction in the protein levels of phosphorylated p65 (p-p65), p65, and CTSK with increasing ZOL concentration, indicating that ZOL suppresses NF-κB activation primarily through inhibition of p65 phosphorylation. Given the regulatory association between PDCD4 and p-p65 [45, 46], we examined upstream regulators using the StarBase database (Fig. 1F). miR-21 was identified as a candidate microRNA targeting PDCD4 and previously linked to osteoclast and osteoblast activity [47, 48]. Functional validation showed that ZOL decreased miR-21 levels while upregulating PDCD4 expression at both mRNA and protein levels (Fig. 1G–H), implying a potential miR-21/PDCD4/p65 axis involved in ZOL-mediated osteoclast inhibition.

### miR-21 enhances osteoclast function and counteracts ZOL-induced suppression via PDCD4 modulation

**Fig. 2:**
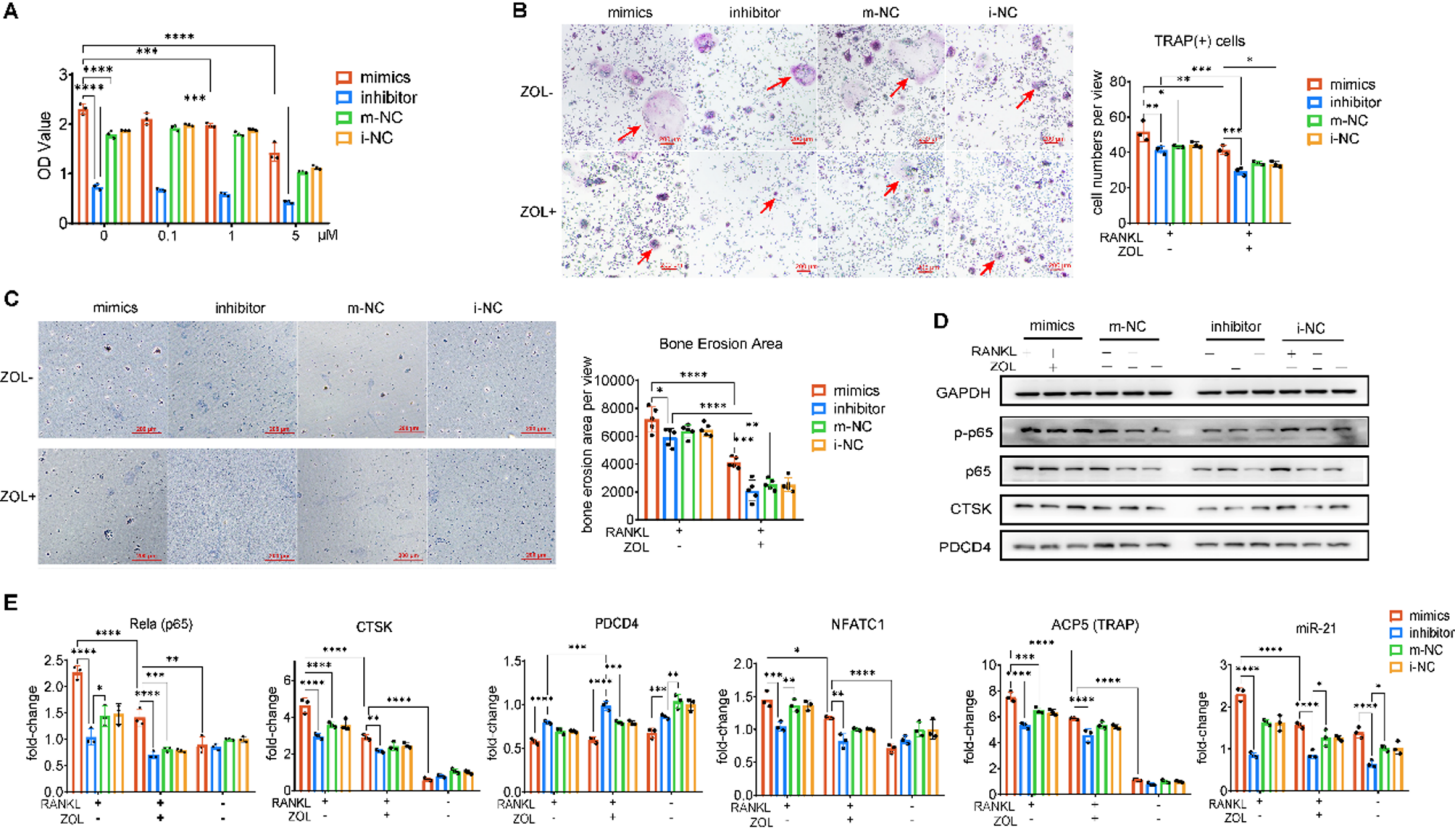
The in vitro biological effects of miR-21 in alleviating inhibitory effect of ZOL on OCs. **A** CCK-8 assay of RAW 264.7 after 24 h of cocultured with RANKL (75 ng/ml) and ZOL at various concentration (0, 0.1, 1, 5 μM); **B** TRAP staining assay and quantitative analysis showed OCs amounts in all groups cultured with ZOL (1 μM); **C** Bone resorption assay assessed OCs bone resorption capacity; **D**, **E** Western blot and RT-qPCR assays measuring the related mRNA and protein expression in different groups. Data are presented as mean ± SD. Statistical significance was determined using one-way ANOVA followed by Tukey’s post hoc test. *P < 0.05; **P < 0.01; ***P < 0.001; ****P < 0.0001

The ability of miR-21 to alleviate ZOL-induced inhibition of osteoclast (OC) function was validated by transfecting RAW 264.7 cells with miR-21 mimics, miR-21 inhibitor, and corresponding negative control (NC) sequences. Cell viability decreased with increasing concentration of ZOL (0, 0.1, 1, and 5 μM). The viability of the mimics group was significantly higher than the m-NC group and the i-NC group, while the inhibitor group showed the worst cell viability (Fig. 2A). Besides, the viability of every group was significantly decreased when the concentration of ZOL was higher than 1 μM. Therefore, ZOL at a concentration of 0–1 μM was considered as the safe concentration range, and the concentration of 1 μM was formulated as the standard for subsequent experiments. TRAP staining indicated that miR-21 overexpression significantly enhanced OC differentiation, as shown by higher numbers of TRAP (+) multinucleated cells in the mimics group compared to other groups (Fig. 2B). Under ZOL treatment, OC formation was suppressed across all groups, yet the mimics group retained a relatively higher differentiation capacity. Consistently, bone resorption assays demonstrated the largest resorption pits in the mimics group under RANKL stimulation, which remained partially preserved despite ZOL exposure (Fig. 2C). Conversely, the inhibitor group exhibited the most pronounced suppression.

Western blot analysis confirmed that, compared with the negative control group, the levels of p-p65 and CTSK were significantly increased, while PDCD4 expression was decreased in the miR-21 mimic group. In contrast, the inhibitor group exhibited reduced expression of these proteins, and these effects were independent of the presence of ZOL (Fig. 2D). At the molecular level, RT-qPCR results demonstrated that miR-21 and osteoclast-related genes (Rela, Nfatc1, Acp5, and Ctsk) were upregulated by RANKL stimulation but suppressed by ZOL treatment, whereas Pdcd4 showed the opposite expression trend. The mimic group exhibited enhanced expression of osteoclast markers and reduced Pdcd4 levels, even under ZOL treatment, while the inhibitor group displayed the opposite pattern (Fig. 2E). Collectively, these findings indicate that miR-21 promotes osteoclast differentiation and function by downregulating PDCD4 and activating the NF-κB signaling pathway, thereby partially reversing the inhibitory effects of ZOL on osteoclastogenesis.

### Biocompatibility and functional validation of MSN-NH₂ for osteogenic applications

**Fig. 3:**
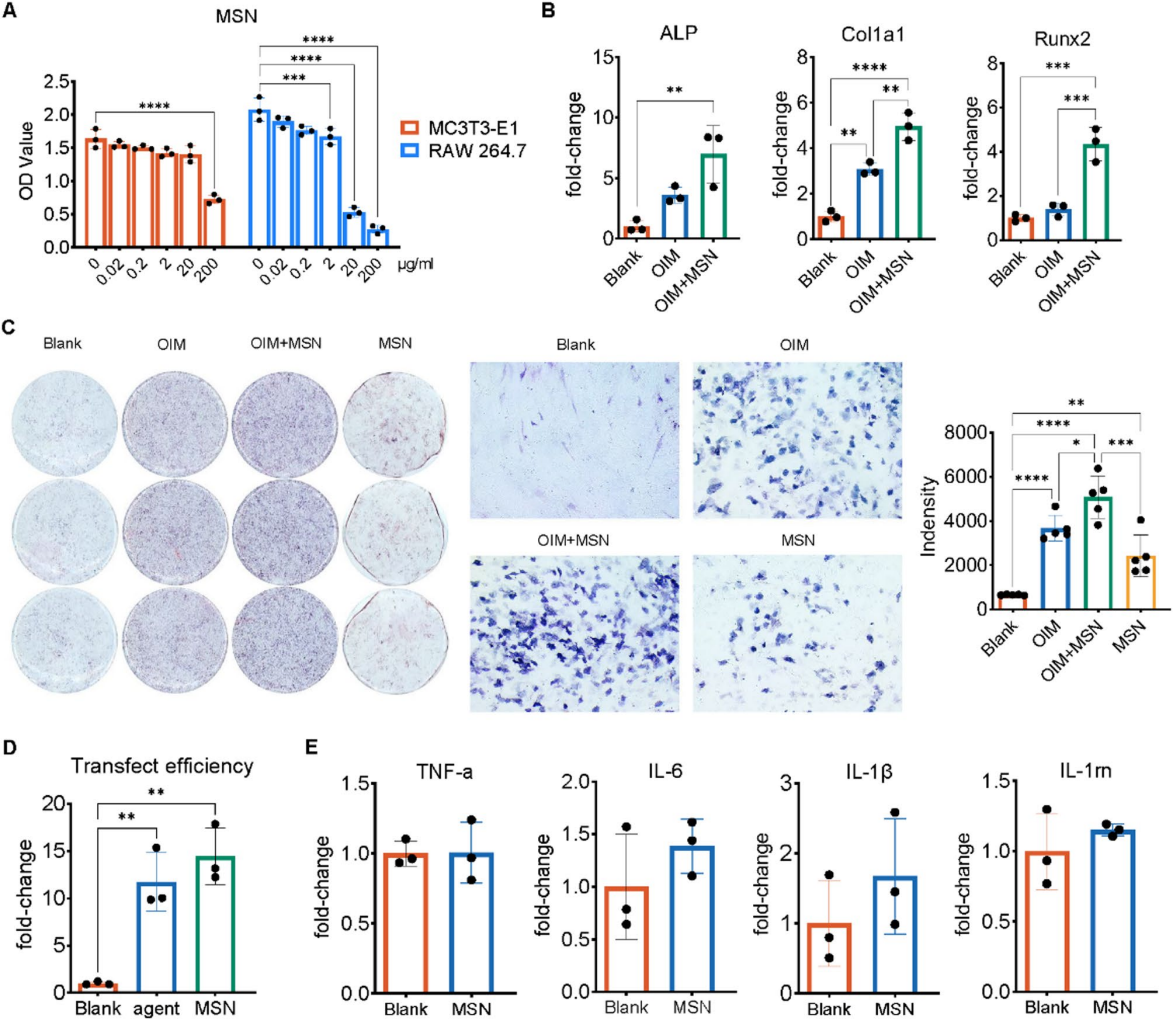
In vitro biological evaluation of MSN-NH₂. **A** Cell viability of MC3T3-E1 and RAW 264.7 cells after 3 days of exposure to varying concentrations of MSN-NH₂, assessed by CCK-8 assay; **B** Osteogenesis genes expression in MC3T3-E1 across different groups were evaluated using RT-qPCR; **C** ALP staining and quantitative analysis of MC3T3-E1 after 7 d of osteogenesis culture; **D** In vitro transfection efficiency of miR-21 to RAW 264.7 was determined by quantifying the miRNA level using RT-qPCR; **E** Inflammation related genes expression in RAW 264.7 with or without MSN were evaluated using RT-qPCR. Data are presented as mean ± SD. Statistical significance was determined using one-way ANOVA followed by Tukey’s post hoc test for multi-group comparisons, and unpaired two-tailed Student’s *t*-test for two-group comparisons. *P < 0.05; **P < 0.01; ***P < 0.001; ****P < 0.0001

To evaluate the applicability of MSN-NH₂ in osteogenesis and gene delivery, its cytocompatibility, osteogenic activity, transfection efficiency, and immunogenicity were assessed. CCK-8 assays showed that MSN-NH₂ was well tolerated by MC3T3-E1 cells up to 200 μg/mL, whereas RAW 264.7 cells exhibited reduced viability above 20 μg/mL (Fig. 3A). At a safe concentration of 2 μg/mL, co-treatment with OIM and MSN significantly upregulated osteogenic markers (ALP, Runx2, and Col1a1) at the mRNA level (Fig. 3B) and enhanced ALP staining intensity (Fig. 3C), indicating its osteoinductive potential. Furthermore, MSN-NH₂ showed comparable miR-21 transfection efficiency to commercial reagents in RAW 264.7 cells (Fig. 3D). Importantly, MSN-NH₂ did not elicit significant increases in pro-inflammatory cytokines (TNF-α, IL-6, IL-1β, IL-1rn), confirming its low immunogenicity (Fig. 3E).

Additionally, to investigate whether MSN alone exerted any influence on osteoclast differentiation or NF-κB/PDCD4 signaling, RAW264.7 cells were treated with MSN+miR-21, miR-21 mimics, or blank control. RT-qPCR analysis showed that the expression levels of osteoclast-related genes, including Rela (p65), Nfatc1, Acp5 (TRAP), and Ctsk, were markedly upregulated in both the MSN+miR-21 and miR-21 groups compared to the blank group, with the MSN+miR-21 group showing the most pronounced increase. In contrast, Pdcd4 expression was significantly downregulated, while miR-21 expression was elevated correspondingly (Supplementary Fig. 1A). Western blot results were consistent with the mRNA data, showing enhanced phosphorylation of p65 and increased CTSK expression, accompanied by reduced PDCD4 levels in the MSN+miR-21 and miR-21 groups, whereas the blank group exhibited minimal activation (Supplementary Fig. 1B). Furthermore, TRAP staining demonstrated that the number of TRAP ( +) multinucleated cells was significantly higher in the MSN+miR-21 and miR-21 groups compared to the blank group, with the MSN+miR-21 group exhibiting the strongest osteoclastogenic activity (Supplementary Fig. 1C-D). Collectively, these findings indicate that MSN alone had negligible effects on osteoclast differentiation and NF-κB/PDCD4 signaling, while miR-21, particularly in MSN-mediated delivery, played a dominant role in promoting osteoclastogenesis.

### Structural characterization and self-healing properties of the Gel/MSN-NH₂ drug delivery system

**Fig. 4:**
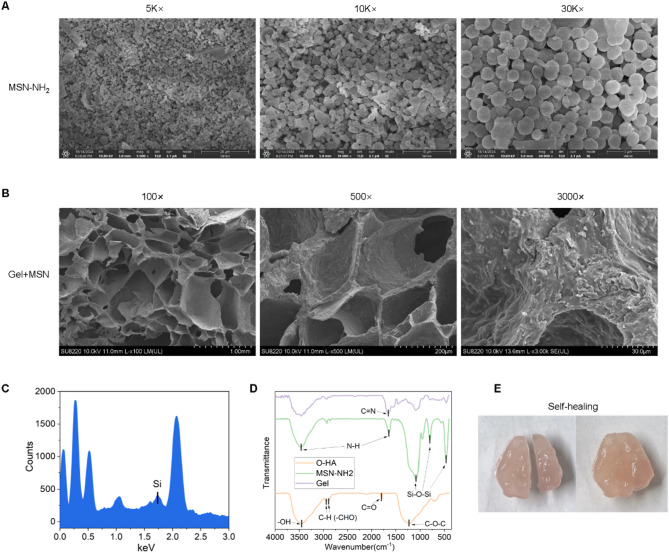
Characterization of MSN-NH₂ and Gel/MSN composite hydrogel. **A** Scanning electron microscopy (SEM) images of MSN-NH₂ at different magnifications (5 K× , 10 K× , and 30 K×) showing uniform spherical morphology with rough surfaces and an average diameter of approximately 600 nm; **B** SEM (100× , 500× , and 3000×) of Gel/MSN+miR-21, showing the MSN-NH_2_ particles distributed on the surface of the hydroge; **C** Energy-dispersive X-ray spectroscopy (EDS) confirming the presence of silicon (Si) peaks, further validating the integration of MSN-NH₂ within the hydrogel system; **D** Fourier-transform infrared (FTIR) spectra of O-HA, MSN-NH₂, Gel, and Gel/MSN composite showing characteristic peaks of each component and the formation of C=N bonds, suggesting successful Schiff base crosslinking between aldehyde and amino groups; **E** Representative images demonstrating the self-healing property of the Gel/MSN hydrogel, which allows the material to restore its integrity after being physically cut, reflecting its dynamic reversible crosslinking nature

Scanning electron microscopy (SEM) analysis demonstrated that MSN-NH₂ particles exhibited a uniform spherical morphology with rough surfaces and an average diameter of approximately 600 nm, confirming their well-defined mesoporous structure (Fig. 4A). Within the hydrogel system, oxidized hyaluronic acid (O–HA) introduced aldehyde groups (–CHO) that reacted with amino groups (–NH₂) from gelatin and MSN-NH₂ through reversible Schiff base bonds to form a stable crosslinked network. SEM images of the lyophilized Gel/MSN composite revealed a homogeneous and interconnected three-dimensional porous architecture, with evenly distributed spherical nanoparticles embedded within the matrix, indicating the successful incorporation of MSN into the hydrogel framework (Fig. 4B). Energy-dispersive X-ray spectroscopy (EDS) confirmed the presence of silicon (Si), further validating the integration of MSN-NH₂ into the gel (Fig. 4C). Fourier-transform infrared (FTIR) analysis revealed characteristic peaks of each component: –C=O– stretching of O–HA at 1720–1740 cm⁻^1^; Si–O–Si of MSN-NH₂ at ~ 440–1080 cm⁻^1^; and N–H vibrations at 3300–3500 and 1550–1650 cm⁻^1^. In the composite system, the disappearance of the –C=O– peak and emergence of a –C=N– peak (1600–1690 cm⁻^1^) indicated successful Schiff base formation (Fig. 4D). Moreover, benefiting from these dynamic covalent bonds, the Gel/MSN hydrogel exhibited excellent self-healing behavior, allowing the material to restore its structural integrity after mechanical disruption and adapt to irregular defect geometries while effectively retaining the MSN-NH₂/miR-21 complex (Fig. 4E). Collectively, these results demonstrate that the Gel/MSN composite hydrogel possesses well-defined morphology, chemical crosslinking, and favorable self-healing capability, providing a stable and functional platform for biomedical applications.

### Gel/MSN+miR-21 promotes bone regeneration in a rat model of MRONJ

**Fig. 5:**
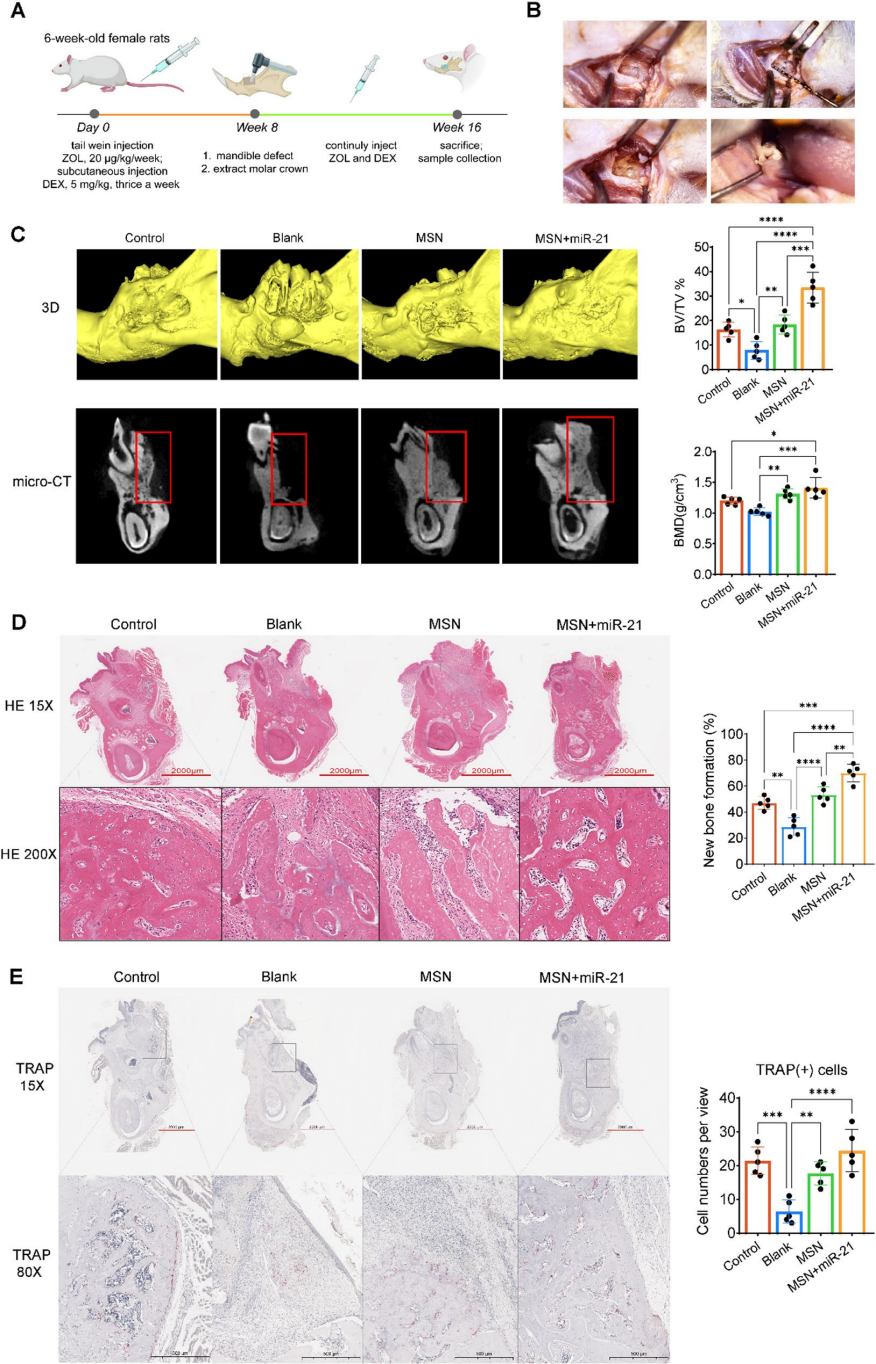
In vivo evaluation of MSN-based miR-21 hydrogel in MRONJ rat model. **A** Schematic illustration of the experimental design for the ZOL+DEX-induced MRONJ model and treatment schedule; **B** Representative intraoperative images showing mandibular defect creation and material implantation; **C** 3D reconstruction and micro-CT cross-sections of mandibular bone in different groups (Control, Blank, MSN, MSN+miR-21). Quantitative analysis of bone volume fraction (BV/TV) and bone mineral density (BMD) revealed significantly improved bone regeneration in the MSN+miR-21 group; **D** HE staining images at low (15×) and high (200 ×) magnifications showing histological bone regeneration within the defect area. Quantification of new bone formation (%) demonstrated enhanced bone repair in the MSN+miR-21 group compared with other groups; **E** TRAP staining showing osteoclast distribution within the defect region; quantification of TRAP( +) cells per view indicated partial restoration of osteoclast activity in the MSN+miR-21 group. Data are presented as mean ± SD (n = 5). One-way ANOVA followed by Tukey’s post hoc test was used for multiple-group comparisons. *P < 0.05; **P < 0.01; ***P < 0.001; ****P < 0.0001

Figure 5A illustrates the experimental design and treatment protocol for establishing the MRONJ rat model. Specifically, ZOL was administered via tail vein injection and DEX was given subcutaneously starting at week 0 for eight consecutive weeks to induce MRONJ. At week 8, mandibular bone defects were created, and molar crowns were extracted. Subsequently, preformed Gel/MSN+miR-21 composite hydrogels were prepared in vitro and implanted into the bone defect sites in equal volumes according to experimental grouping. ZOL and DEX administration continued until week 16, when the rats were sacrificed, and mandibular samples were collected for analysis. Notably, Fig. 5B presents representative intraoperative images showing the creation of mandibular defects, molar crown extraction, and placement of the hydrogel within the defect site. We observed that the Blank group exhibited pronounced bone loss and trabecular rarefaction within the defect area, while the MSN group showed mild bone formation. In contrast, the MSN+miR-21 group displayed denser, more continuous trabecular structures and markedly higher bone mineral density (Fig. 5C). Quantitative analysis confirmed that both BV/TV and BMD were significantly increased in the MSN+miR-21 group compared with the other groups. HE staining further supported these findings (Fig. 5D). the Blank group showed extensive fibrous tissue filling within the defect, the MSN group exhibited limited and discontinuous new bone formation, whereas the MSN+miR-21 group presented abundant, well-organized new bone plates and trabeculae that integrated seamlessly with the host bone. Additionally, TRAP staining revealed a significant reduction of osteoclast numbers in the Blank group, indicating suppressed bone resorption induced by ZOL treatment. Notably, the MSN+miR-21 group demonstrated a partial restoration of osteoclast numbers, suggesting that miR-21 delivery enhanced osteoclast activity and promoted bone remodeling (Fig. 5E). In addition, RISH analysis demonstrated miR-21 expression in mandibular bone tissues of each group. The Blank group showed weak and scattered miR-21-positive signals around the defect area, the MSN group exhibited moderate enhancement, whereas the MSN+miR-21 group displayed the strongest and most widespread signals, mainly localized in newly formed bone and surrounding cells (Supplementary Fig. 2). Collectively, these results indicate that miR-21-loaded MSN hydrogel effectively promotes bone repair and regeneration in the MRONJ model.

### The Gel/MSN+miR-21 system exerts localized effects on the mandible without impairing systemic bone metabolism or major organ integrity

**Fig. 6:**
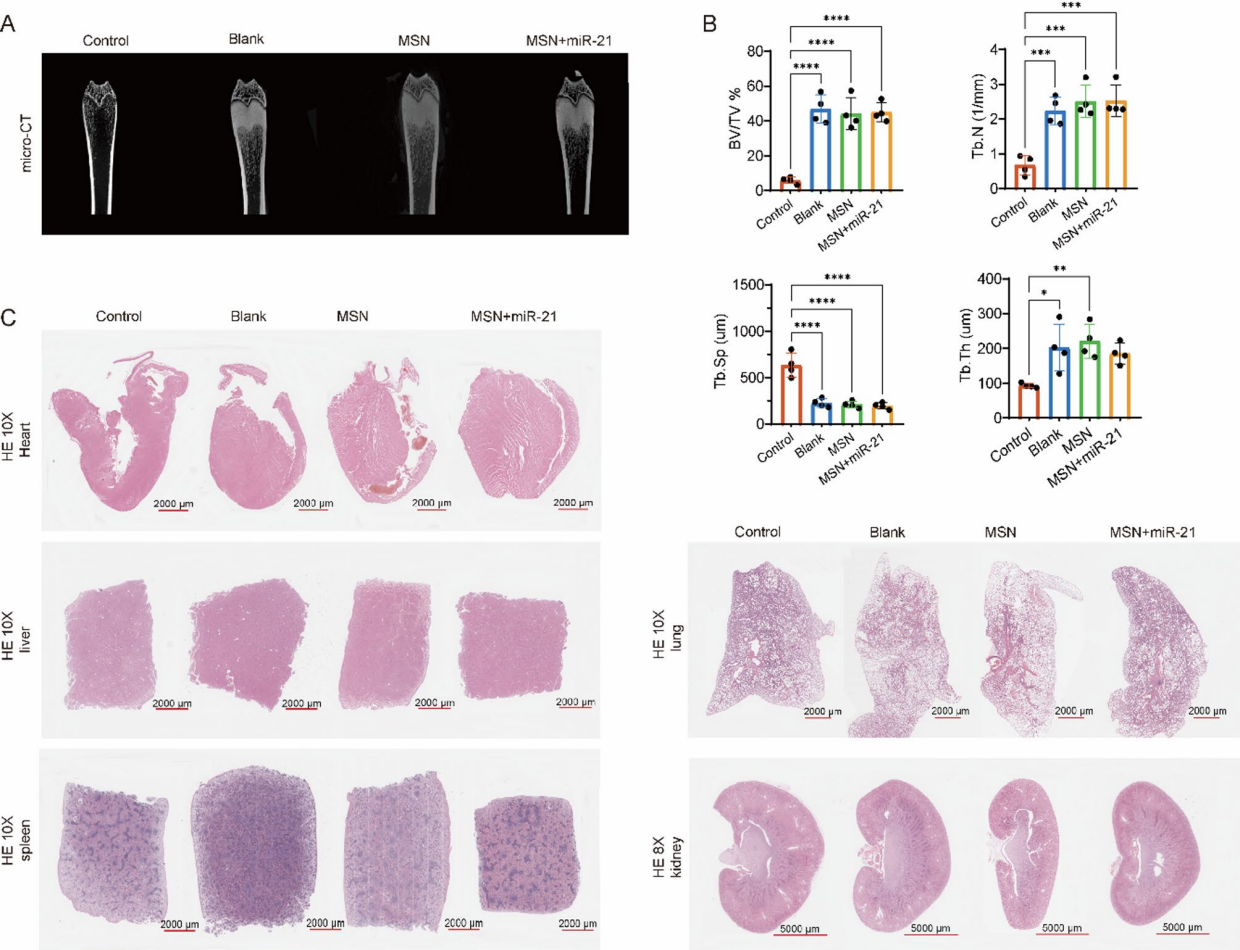
Evaluation of bone regeneration and systemic biosafety in vivo. **A** Representative in vivo micro-CT images of femurs from each group (Control, Blank, MSN, and MSN+miR-21). The reconstructed cross-sectional views show overall bone morphology, confirming no gross skeletal abnormalities or systemic adverse effects after local treatment; **B** Quantitative analysis of trabecular microarchitecture based on micro-CT data, including bone volume fraction (BV/TV), trabecular number (Tb.N), trabecular separation (Tb.Sp), and trabecular thickness (Tb.Th); **C** Hematoxylin and eosin (HE) staining of major organs (heart, liver, spleen, lung, and kidney) from each group revealed no noticeable histopathological abnormalities. Data are expressed as mean ± SD. Statistical significance was assessed by one-way ANOVA followed by Tukey’s post hoc test for multiple comparisons. * *P* < 0.05; ** *P* < 0.01; *** *P* < 0.001; **** *P* < 0.0001

To assess the systemic safety and potential effects of the miR-21-loaded MSN hydrogel on femoral bone structure, micro-CT analysis demonstrated that the overall morphology of femurs remained intact across all groups, with no observable bone defects or abnormal remodeling (Fig. 6A). As shown in Fig. 6B, compared with the untreated control group, ZOL administration in the Blank, MSN, and MSN+miR-21 groups significantly increased trabecular bone volume fraction (BV/TV), number (Tb.N), and thickness (Tb.Th), while decreasing trabecular separation (Tb.Sp). HE staining revealed well-preserved bone microarchitecture in all groups (Supplementary Fig. 3). Notably, histological evaluation of major organs (heart, liver, spleen, lung, and kidney) showed intact tissue morphology without inflammation or necrosis (Fig. 6C), indicating that local administration of the MSN+miR-21 hydrogel did not induce systemic toxicity or bone structural damage, supporting its favorable biosafety and translational potential.

## Discussion

MRONJ is a rare but serious adverse effect associated with anti-resorptive therapies such as bisphosphonates (BPs), and its pathogenesis remains incompletely understood [49]. Current evidence suggests that dysregulated osteoclast activity and impaired bone remodeling are central to MRONJ development [50, 51]. For instance, miR-21-5p has been found to be upregulated during the early healing phase of MRONJ lesions, implicating its involvement in the altered bone repair response [52]. Additionally, extracellular vesicles (EVs) derived from zoledronate (ZA)-treated OCs were shown to carry elevated levels of miRNAs such as miR-146a-5p and miR-322-3p, which respectively suppress osteoclast and influence osteoblast differentiation, highlighting the multifaceted effects of ZA on bone cellular crosstalk [53–55]. However, whether specific miRNAs such as miR-21 can be harnessed to reverse ZOL-induced inhibition of osteoclast function and promote bone regeneration remains largely unexplored. In the present study, we demonstrated that miR-21 alleviates ZOL-induced suppression of osteoclastogenesis by targeting PDCD4, restoring p65 phosphorylation and downstream NF-κB signaling. Furthermore, we developed a preformed hydrogel (Gel/MSN+miR-21) capable of delivering miR-21 to the mandible, reestablishing osteoclast–osteoblast coupling, and significantly enhancing bone regeneration in a rat model of MRONJ. These findings uncover a new regulatory mechanism and provide a targeted therapeutic strategy for MRONJ management.

MiR-21 is now gaining increasing recognition as a molecule that regulates bone tissue homeostasis [17, 56]. On one hand, miR-21 promotes osteogenic differentiation and mineralization by modulating factors such as Runx2, OPN, and RANKL, while also maintaining osteoclast activity through paracrine signaling [57]. On the other hand, miR-21 can regulate osteoclast survival and apoptosis by responding to lncRNA GAS5, thus positively influencing bone resorption [58]. Additionally, research has shown that miR-21-loaded nanocapsules on titanium surfaces can simultaneously enhance angiogenesis, osteogenesis, and osteoclastogenesis, thereby accelerating osseointegration [59, 60]. Collectively, these findings highlight the dual regulatory function of miR-21 in promoting both bone formation and bone resorption, making it a pivotal molecule for dynamic bone remodeling. Although miR-21 has demonstrated promising osteogenic and osteoclastic regulatory effects in bone regeneration, its potential long-term risks warrant further consideration. MiR-21 is a multifunctional microRNA that regulates a broad spectrum of target genes involved in apoptosis, inflammation, and cell differentiation [61]. Consequently, off-target gene silencing may occur, particularly under conditions of sustained or excessive miR-21 expression. For example, miR-21 has been reported to downregulate PTEN, PDCD4, and RECK—tumor suppressors that are essential for maintaining cellular homeostasis—thereby potentially promoting abnormal cell proliferation and migration in non-target tissues [62, 63]. Moreover, miR-21 plays a dual role in cancer biology, acting as an oncogene in hepatocellular carcinoma, breast cancer, and prostate cancer, but exhibiting tumor-suppressive effects in certain immune or stromal contexts depending on cellular background and microenvironmental cues [64–66]. This underscores the complexity of miR-21 regulation and the need for careful dose control and spatial restrictions when designing therapeutic strategies. In addition to oncogenic risks, miR-21 also exerts profound effects on immune modulation. It has been shown to promote M2 macrophage polarization and suppress pro-inflammatory cytokine expression through regulation of the NF-κB and STAT3 pathways [67–69]. Although these anti-inflammatory properties may benefit tissue regeneration and wound healing, excessive immunosuppression could impair host defense mechanisms or alter local immune homeostasis. Therefore, the long-term immunological consequences of miR-21 delivery should be carefully assessed in future in vivo studies. Importantly, the localized MSN-based hydrogel platform used in this study provides spatially confined delivery, minimizing systemic exposure and reducing the likelihood of off-target or oncogenic effects. Nevertheless, future research should systematically evaluate the degradation timeline, tissue distribution, and biosafety of miR-21 in extended models to confirm its therapeutic window and avoid potential adverse outcomes.

Previous studies have often established MRONJ mandibular defect models in the mandibular ramus, a region that does not directly communicate with the oral extraction sites, or by extracting the first molar with or without introducing specific microbiota [70–72]. However, these approaches may not accurately replicate the clinical condition of unhealed extraction sockets or the complex microenvironment following curettage of necrotic jawbone in MRONJ patients. In this study, we established a novel MRONJ bone defect model by creating defects in the mandibular molar region that included the roots of the first molar, while the tooth crown was removed and the roots were retained within the defect area. This approach closely mimics clinical scenarios where the extraction site fails to heal and the alveolar bone remains exposed to the oral cavity. Additionally, since the rats were raised under standard laboratory conditions without microbiota control, their oral environment maintained a natural complexity, further enhancing the clinical relevance of the model. Compared to existing models, our design better reflects the pathophysiological features of MRONJ and provides a more suitable platform for evaluating the therapeutic efficacy of localized drug delivery systems such as the Gel/MSN+miR-21 hydrogel used in this study. In the rat MRONJ model established in this study, a significant periosteal reaction (PR) was observed, characterized by new bone formation along the cortical surface. PR is typically associated with chronic inflammatory stimuli and is recognized as a radiographic feature indicative of poor prognosis in MRONJ patients [73]. It can be classified into three types: attached-type, gap-type, and irregular-type, with the latter two often linked to infectious lesions such as bacterial or fungal infections [74]. Unlike the proliferative PR observed in osteomyelitis, PR in MRONJ tends to be more destructive and is thought to result from osteoclast apoptosis and subsequent bone remodeling disturbances, particularly under high-dose anti-resorptive treatment [73]. In our model, both attached-type and gap-type PR were evident, likely due to the communication between the exposed residual molar roots and the defect site, facilitating persistent bacterial invasion. Importantly, despite the presence of substantial PR and a challenging inflammatory microenvironment, the locally applied Gel/MSN+miR-21 hydrogel system demonstrated excellent regenerative capacity. It significantly promoted bone defect healing without signs of persistent bone exposure, oral fistulas, or extraoral infections, highlighting its therapeutic potential in clinically relevant MRONJ conditions.

Notably, in this study, the hydrogel matrix is primarily formed via dynamic Schiff base linkages between O-HA and gelatin, enabling gradual hydrolysis and reversible bond cleavage under physiological conditions. Such dynamic covalent networks endow the system with controlled biodegradability, typically within a period of 2–4 weeks depending on the crosslinking density and enzymatic activity of the local environment [75, 76]. Previous studies have demonstrated that O-HA/Gel-based hydrogels undergo progressive softening and partial dissolution during the bone remodeling period, which aligns well with the tissue healing timeline [77]. The incorporation of MSN slightly prolongs the degradation rate due to their stable Si–O–Si framework, allowing sustained mechanical integrity and continuous local release of miR-21 throughout the repair process [78, 79]. Regarding the release kinetics of miR-21, MSN acts as a reservoir that provides both physical confinement and electrostatic adsorption for miRNA molecules, enabling stepwise release as the gel network gradually disassembles. This hybrid structure ensures a dual-phase release pattern—an initial mild burst followed by a sustained release over several weeks—which is consistent with previous reports of MSN-loaded polysaccharide hydrogels used in bone tissue regeneration [79]. The observed biological effects in vitro and in vivo, including enhanced osteoclast differentiation and improved bone healing within the MRONJ model, indirectly confirm that the miR-21 remained bioactive and was released in a temporally controlled manner. Future studies will include systematic characterization of degradation kinetics, rheological evolution, and cumulative miRNA release curves to further elucidate the relationship between the hydrogel degradation rate, miRNA stability, and therapeutic duration.

In clinical settings, especially among patients with conditions such as breast cancer bone metastases, high-dose anti-resorptive drugs like bisphosphonates or denosumab are routinely administered to control tumor-induced osteolysis and skeletal-related events. Although these patients are at a significantly elevated risk of developing MRONJ, systemic anti-resorptive therapy remains indispensable for managing their primary disease. Premature discontinuation or unintended interference with the systemic effects of these drugs could lead to serious consequences, including pathological fractures or cancer progression [80]. Therefore, in developing local therapeutic strategies for MRONJ, it is critical to ensure that the intervention remains localized and does not compromise the systemic pharmacodynamics of anti-resorptive agents. Another limitation of this study is that all in vivo experiments were performed in a rodent MRONJ model, which, although well-established and reproducible, may not fully replicate the complex pathophysiological features of human MRONJ. Differences in bone microarchitecture, drug metabolism, immune response, and healing capacity between rodents and humans may influence the translational relevance of the findings. Therefore, while the Gel/MSN+miR-21 system demonstrated significant therapeutic efficacy in rats, caution should be exercised when extrapolating these results to clinical applications. Future studies will aim to validate the safety, pharmacodynamics, and efficacy of this system in large animal models or in ex vivo human jawbone tissue cultures. In addition, this study did not include a systematic observation or quantitative analysis of necrotic bone formation. In future work, we plan to extend the observation period and employ large animal models that better mimic clinical pathology, incorporating analyses such as the proportion of empty lacunae and measurement of necrotic bone area to systematically investigate the formation and progression of necrotic bone. Such investigations will provide a more comprehensive preclinical foundation to support potential clinical translation. Finally, this study did not include rheological analyses of the hydrogel system under different compositional conditions, namely the pure hydrogel, Gel+MSN, and Gel+MSN+miR-21 formulations. Consequently, quantitative characterization of viscoelastic parameters was not performed, which may partially limit our understanding of the crosslinking network density, dynamic plasticity, and structural stability of the system. In future work, we plan to incorporate comprehensive rheological measurements using a rotational rheometer to evaluate hydrogels with varying component ratios. These experiments will be complemented by FTIR, DSC, and swelling analyses to elucidate the structure–property relationships of the material. Through these additional studies, we aim to further clarify the roles of MSN and miR-21 in modulating the hydrogel network architecture and stability, thereby providing a more robust physicochemical basis for the controllability and engineering optimization of this localized drug delivery system.

## Conclusions

This study demonstrated that ZOL inhibited osteoclast function by upregulating PDCD4 and suppressing NF-κB (p65) phosphorylation. miR-21 was identified to target PDCD4, restoring p65 activation and rescuing ZOL-induced suppression of OCs differentiation and bone resorption. Based on this mechanism, a miR-21-loaded MSN hydrogel was developed, which enabled sustained miR-21 release, promoted new bone formation, and enhanced bone remodeling in vivo. Overall, local delivery of miR-21 exhibited favorable bone repair potential and biosafety, providing experimental evidence for a novel therapeutic strategy in MRONJ.

## Supplementary Information

Below is the link to the electronic supplementary material.

Supplementary file 1.

Supplementary file 2.

Supplementary file 3.

Supplementary file 4.

Supplementary file 5.

## Data Availability

Data will be made available on request. See the "Supplementary material" file for the complete uncut gel and imprint images of this study.

## References

[1] Lee K, Kim K, Kim JY, et al. Mechanisms underlying medication-related osteonecrosis of the jaw. Oral Dis. 2025;31(4):1073–83. 39552606 10.1111/odi.15198 PMC12022389

[2] Murphy J, Mannion CJ. Medication-related osteonecrosis of the jaws and quality of life: review and structured analysis. Br J Oral Maxillofac Surg. 2020;58(6):619–24. 32247520 10.1016/j.bjoms.2020.03.010

[3] Marx RE. Pamidronate (Aredia) and zoledronate (Zometa) induced avascular necrosis of the jaws: a growing epidemic. J Oral Maxillofac Surg. 2003;61(9):1115–7. 12966493 10.1016/s0278-2391(03)00720-1

[4] Patel N, Seoudi N. Management of medication-related osteonecrosis of the jaw: an overview of national and international guidelines. Br J Oral Maxillofac Surg. 2024;62(10):899–908. 39448352 10.1016/j.bjoms.2024.08.008

[5] Stellato M, Zecca E, Bracchi P, et al. Medication-related jaw osteonecrosis in metastatic RCC treated with VEGFR-TKIs ± IO and bone agents: a real-world analysis. Tumori. 2025. 10.1177/03008916251363754. 40878642 10.1177/03008916251363754

[6] Bassan Marinho Maciel G, Marinho Maciel R, Linhares Ferrazzo K, Cademartori Danesi C. Etiopathogenesis of medication-related osteonecrosis of the jaws: a review. J Mol Med. 2024;102(3):353–64. 38302741 10.1007/s00109-024-02425-9

[7] Huang F, Wang Y, Liu J, Cheng Y, Zhang X, Jiang H. Asperuloside alleviates osteoporosis by promoting autophagy and regulating Nrf2 activation. J Orthop Surg Res. 2024;19(1):855. 39702357 10.1186/s13018-024-05320-8 PMC11658297

[8] Shen L, Yang H, Zhou F, Jiang T, Jiang Z. Risk factors of short-term residual low back pain after PKP for the first thoracolumbar osteoporotic vertebral compression fracture. J Orthop Surg Res. 2024;19(1):792. 39587591 10.1186/s13018-024-05295-6 PMC11590304

[9] Leeyaphan J, Rojjananukulpong K, Intarasompun P, Peerakul Y. Simple clinical predictors for making directive decisions in osteoporosis screening for women: a cross-sectional study. J Orthop Surg Res. 2024;19(1):789. 39581985 10.1186/s13018-024-05287-6 PMC11585944

[10] Zhang D, Zuo M, Zhou J, et al. A facile combined therapy of chemotherapeutic agent and microRNA for hepatocellular carcinoma using non-cationic nanogel. J Mater Chem B. 2025;13(8):2753–66. 39868422 10.1039/d4tb02256d

[11] Giordano L, Porta GD, Peretti GM, Maffulli N. Therapeutic potential of microrna in tendon injuries. Br Med Bull. 2020;133(1):79–94. 32219416 10.1093/bmb/ldaa002

[12] Gargano G, Oliviero A, Oliva F, Maffulli N. Small interfering RNAs in tendon homeostasis. Br Med Bull. 2021;138(1):58–67. 33454750 10.1093/bmb/ldaa040

[13] Oliviero A, Della Porta G, Peretti GM, Maffulli N. Microrna in osteoarthritis: physiopathology, diagnosis and therapeutic challenge. Br Med Bull. 2019;130(1):137–47. 31066454 10.1093/bmb/ldz015

[14] Gargano G, Oliva F, Oliviero A, Maffulli N. Small interfering RNAs in the management of human rheumatoid arthritis. Br Med Bull. 2022;142(1):34–43. 35488320 10.1093/bmb/ldac012 PMC9351475

[15] Gargano G, Asparago G, Spiezia F, Oliva F, Maffulli N. Small interfering RNAs in the management of human osteoporosis. Br Med Bull. 2023;148(1):58–69. 37675799 10.1093/bmb/ldad023 PMC10788844

[16] Migliorini F, Maffulli N, Spiezia F, Tingart M, Maria PG, Riccardo G. Biomarkers as therapy monitoring for postmenopausal osteoporosis: a systematic review. J Orthop Surg Res. 2021;16(1):318. 34006294 10.1186/s13018-021-02474-7 PMC8130375

[17] Migliorini F, Maffulli N, Spiezia F, Peretti GM, Tingart M, Giorgino R. Potential of biomarkers during pharmacological therapy setting for postmenopausal osteoporosis: a systematic review. J Orthop Surg Res. 2021;16(1):351. 34059108 10.1186/s13018-021-02497-0 PMC8165809

[18] Yalaev BI, Kaletnik EI, Karpova YS, et al. The role of microRNA in the regulation of differentiation and the functionality of osteoblasts, osteoclasts, and their precursors in osteoporosis. Non-coding RNA. 2025;11(1):14. 39997614 10.3390/ncrna11010014 PMC11858178

[19] Mohd Yunus SS, Soh HY, Abdul Rahman M, Peng X, Guo C, Ramli R. MicroRNA in medication related osteonecrosis of the jaw: a review. Front Physiol. 2023;14:1021429. 37179831 10.3389/fphys.2023.1021429 PMC10169589

[20] Santini D, Vincenzi B, Dicuonzo G, et al. Zoledronic acid induces significant and long-lasting modifications of circulating angiogenic factors in cancer patients. Clin Canc Res: Off J Am Associat Canc Res. 2003;9(8):2893–7. 12912933

[21] Allegra A, Alonci A, Penna G, et al. Bisphosphonates induce apoptosis of circulating endothelial cells in multiple myeloma patients and in subjects with bisphosphonate-induced osteonecrosis of the jaws. Acta Haematol. 2010;124(2):79–85. 20639624 10.1159/000313787

[22] Oteri G, Allegra A, Bellomo G, et al. Reduced serum levels of interleukin 17 in patients with osteonecrosis of the jaw and in multiple myeloma subjects after bisphosphonates administration. Cytokine. 2008;43(2):103–4. 18585926 10.1016/j.cyto.2008.05.010

[23] Chen C, Liu YM, Fu BL, Xu LL, Wang B. Microrna-21: an emerging player in bone diseases. Front Pharmacol. 2021;12:722804. 34557095 10.3389/fphar.2021.722804 PMC8452984

[24] Niazi SK, Magoola M. MicroRNA Nobel Prize: Timely Recognition and High Anticipation of Future Products-A Prospective Analysis. Int J Mol Sci. 2024. 10.3390/ijms252312883. 39684593 10.3390/ijms252312883 PMC11641023

[25] Cha W, Fan R, Miao Y, et al. Mesoporous Silica Nanoparticles as Carriers for Intracellular Delivery of Nucleic Acids and Subsequent Therapeutic Applications. Molecules (Basel, Switzerland) 2017, 22(5). 10.3390/molecules22050782 PMC6154527 28492505

[26] Shadjou N, Hasanzadeh M. Silica-based mesoporous nanobiomaterials as promoter of bone regeneration process. J Biomed Mater Res A. 2015;103(11):3703–16. 26011776 10.1002/jbm.a.35504

[27] Andersen M, Andresen AK, Hartvigsen J, Hermann AP, Sørensen J, Carreon LY. Vertebroplasty for painful osteoporotic vertebral compression fractures: a protocol for a single-center doubled-blind randomized sham-controlled clinical trial. VOPE2. J Orthop Surg Res. 2024;19(1):813. 39614265 10.1186/s13018-024-05301-x PMC11607804

[28] Li Y, Chen X, Jin R, et al. Injectable hydrogel with MSNs/microRNA-21-5p delivery enables both immunomodification and enhanced angiogenesis for myocardial infarction therapy in pigs. Sci Adv. 2021. 10.1126/sciadv.abd6740. 33627421 10.1126/sciadv.abd6740 PMC7904259

[29] Zhu W, Dong Y, Xu P, et al. A composite hydrogel containing resveratrol-laden nanoparticles and platelet-derived extracellular vesicles promotes wound healing in diabetic mice. Acta Biomater. 2022;154:212–30. 36309190 10.1016/j.actbio.2022.10.038

[30] Li H, Zhao T, Yuan Z, et al. Cartilage lacuna-biomimetic hydrogel microspheres endowed with integrated biological signal boost endogenous articular cartilage regeneration. Bioact Mater. 2024;41:61–82. 39104774 10.1016/j.bioactmat.2024.06.037 PMC11299526

[31] Li JH, Liu S, Zhou H, Qu LH, Yang JH. StarBase v2.0: decoding miRNA-ceRNA, miRNA-ncRNA and protein-RNA interaction networks from large-scale CLIP-Seq data. Nucleic Acids Res. 2014;42(Database issue):D92-97. 24297251 10.1093/nar/gkt1248 PMC3964941

[32] Liu Y, Wang W, Zeng Y, Zeng H. Transcriptome analysis of hydrogen inhibits osteoclastogenesis of mouse bone marrow mononuclear cells. Exp Ther Med. 2023;26(3):436. 37614423 10.3892/etm.2023.12135 PMC10443061

[33] Xu S, Zhang Z, Zhou X, et al. Gouqi-derived nanovesicles (GqDNVs) promoted MC3T3-E1 cells proliferation and improve fracture healing. Phytomedicine. 2025;142:156755. 40252435 10.1016/j.phymed.2025.156755

[34] Mamun-Or-Rashid ANM, Lucy TT, Yagi M, Yonei Y. Inhibitory effects of astaxanthin on CML-HSA-induced inflammatory and RANKL-induced osteoclastogenic gene expression in RAW 264.7 cells. Biomedicines. 2021. 10.3390/biomedicines10010054. 35052734 10.3390/biomedicines10010054 PMC8772757

[35] Eckert D, Rapp F, Tsedeke AT, et al. Modulation of differentiation and bone resorbing activity of human (pre-) osteoclasts after X-ray exposure. Front Immunol. 2022;13:817281. 35603191 10.3389/fimmu.2022.817281 PMC9116137

[36] Ren MS, Xie HH, Ding Y, Li ZH, Liu B. Er-xian decoction drug-containing serum promotes Mc3t3-e1 cell proliferation and osteogenic differentiation via regulating BK channel. J Ethnopharmacol. 2023;302(Pt A):115887. 36328203 10.1016/j.jep.2022.115887

[37] Rao X, Huang X, Zhou Z, Lin X. An improvement of the 2ˆ(-delta delta CT) method for quantitative real-time polymerase chain reaction data analysis. Biostatistics, bioinformatics and biomathematics. 2013;3(3):71–85. 25558171 PMC4280562

[38] Mao N, Yu Y, Lu X, Yang Y, Liu Z, Wang D. Preventive effects of matrine on LPS-induced inflammation in RAW 2647 cells and intestinal damage in mice through the TLR4/NF-κB/MAPK pathway. Int Immunopharmacol. 2024;143(Pt 2):113432. 39447411 10.1016/j.intimp.2024.113432

[39] Muhammad M, Willems C, Rodríguez-Fernández J, Gallego-Ferrer G, Groth T. Synthesis and characterization of oxidized polysaccharides for in situ forming hydrogels. Biomolecules. 2020. 10.3390/biom10081185. 32824101 10.3390/biom10081185 PMC7464976

[40] Estevão BM, Miletto I, Hioka N, Marchese L, Gianotti E. Mesoporous silica nanoparticles functionalized with amino groups for biomedical applications. ChemistryOpen. 2021;10(12):1251–9. 34907672 10.1002/open.202100227 PMC8671895

[41] Kozutsumi R, Kuroshima S, Al-Omari FA, et al. Depletion of macrophages deteriorates bisphosphonate-related osteonecrosis of the jaw-like lesions in mice. Bone. 2023;177:116899. 37708951 10.1016/j.bone.2023.116899

[42] Migliorini F, Colarossi G, Baroncini A, Eschweiler J, Tingart M, Maffulli N. Pharmacological management of postmenopausal osteoporosis: a level I evidence based - expert opinion. Expert Rev Clin Pharmacol. 2021;14(1):105–19. 33183112 10.1080/17512433.2021.1851192

[43] Zheng X, Qiu J, Gao N, et al. Paroxetine attenuates chondrocyte pyroptosis and inhibits osteoclast formation by inhibiting NF-κB pathway activation to delay osteoarthritis progression. Drug Des Dev Ther. 2023;17:2383–99. 10.2147/DDDT.S417598 PMC10440089 37605762

[44] Hanna JA, Wimberly H, Kumar S, Slack F, Agarwal S, Rimm DL. Quantitative analysis of microRNAs in tissue microarrays by in situ hybridization. Biotechniques. 2012;52(4):235–45. 22482439 10.2144/000113837 PMC3891915

[45] Yu L, Yang Y, Wang J, et al. PDCD4 promotes inflammation/fibrosis by activating the PPAR‑γ/NF‑κB pathway in mouse atrial myocytes. Mol Med Rep. 2024. 10.3892/mmr.2024.13333. 39301631 10.3892/mmr.2024.13333 PMC11425064

[46] Hwang SK, Baker AR, Young MR, Colburn NH. Tumor suppressor PDCD4 inhibits NF-κB-dependent transcription in human glioblastoma cells by direct interaction with p65. Carcinogenesis. 2014;35(7):1469–80. 24413684 10.1093/carcin/bgu008 PMC4076808

[47] Hu CH, Sui BD, Du FY, et al. MiR-21 deficiency inhibits osteoclast function and prevents bone loss in mice. Sci Rep. 2017;7:43191. 28240263 10.1038/srep43191 PMC5327426

[48] Zhou Y, Liu Y, Cheng L. Mir-21 expression is related to particle-induced osteolysis pathogenesis. J Orthop Res. 2012;30(11):1837–42. 22508494 10.1002/jor.22128

[49] Frutuoso F, Freitas F, Vilares M, et al. Medication-related osteonecrosis of the jaw: a systematic review of case reports and case series. Diseases (Basel, Switzerland). 2024;12(9):205. 39329874 10.3390/diseases12090205 PMC11431443

[50] Migliorini F, Giorgino R, Hildebrand F, et al. Risk factors and management in the elderly. Medicina (Kaunas). 2021;57(10):1119. 34684156 10.3390/medicina57101119 PMC8538459

[51] Conti V, Russomanno G, Corbi G, et al. A polymorphism at the translation start site of the vitamin D receptor gene is associated with the response to anti-osteoporotic therapy in postmenopausal women from southern Italy. Int J Mol Sci. 2015;16(3):5452–66. 25764158 10.3390/ijms16035452 PMC4394486

[52] Morakotsriwan M, Chanamuangkon T, Vacharaksa A, Limlawan P. Microrna-21-5p profile in the alveolar bone following tooth extraction in medication-related osteonecrosis of the jaw rat model. Front Dent Med. 2024;5:1477274. 39917666 10.3389/fdmed.2024.1477274 PMC11797932

[53] Minami S, Fujii Y, Yoshioka Y, et al. Extracellular vesicles from mouse bone marrow macrophages-derived osteoclasts treated with zoledronic acid contain miR-146a-5p and miR-322-3p, which inhibit osteoclast function. Bone. 2025;190:117323. 39510435 10.1016/j.bone.2024.117323

[54] Migliorini F, Colarossi G, Eschweiler J, Oliva F, Driessen A, Maffulli N. Antiresorptive treatments for corticosteroid-induced osteoporosis: a Bayesian network meta-analysis. Br Med Bull. 2022;143(1):46–56. 35641234 10.1093/bmb/ldac017 PMC9494254

[55] Gargano G, Pagano SM, Maffulli N. Circular RNAs in the management of human osteoporosis. Br Med Bull. 2025. 10.1093/bmb/ldae024. 39821210 10.1093/bmb/ldae024

[56] Bao X, Liu C, Liu H, Wang Y, Xue P, Li Y. Association between polymorphisms of glucagon-like peptide-1 receptor gene and susceptibility to osteoporosis in Chinese postmenopausal women. J Orthop Surg Res. 2024;19(1):869. 39716293 10.1186/s13018-024-05361-z PMC11667832

[57] Smieszek A, Marcinkowska K, Pielok A, Sikora M, Valihrach L, Marycz K. The role of miR-21 in osteoblasts-osteoclasts coupling in vitro. Cells. 2020. 10.3390/cells9020479. 32093031 10.3390/cells9020479 PMC7072787

[58] Cong C, Tian J, Gao T, et al. LncRNA GAS5 Is upregulated in osteoporosis and downregulates miR-21 to promote apoptosis of osteoclasts. Clin Interv Aging. 2020;15:1163–9. 32764903 10.2147/CIA.S235197 PMC7371557

[59] Geng Z, Yu Y, Li Z, et al. Mir-21 promotes osseointegration and mineralization through enhancing both osteogenic and osteoclastic expression. Mater Sci Eng C Mater Biol Appl. 2020;111:110785. 32279740 10.1016/j.msec.2020.110785

[60] Migliorini F, Maffulli N, Colarossi G, Eschweiler J, Tingart M, Betsch M. Effect of drugs on bone mineral density in postmenopausal osteoporosis: a Bayesian network meta-analysis. J Orthop Surg Res. 2021;16(1):533. 34452621 10.1186/s13018-021-02678-x PMC8393477

[61] Syed RU, Banu H, Alshammrani A, et al. Microrna-21 (miR-21) in breast cancer: from apoptosis dysregulation to therapeutic opportunities. Pathology. 2024;262:155572. 10.1016/j.prp.2024.155572 39226804

[62] He Z, Long J, Yang C, et al. LncRNA DGCR5 plays a tumor-suppressive role in glioma via the miR-21/Smad7 and miR-23a/PTEN axes. Aging. 2020;12(20):20285–307. 33085646 10.18632/aging.103800 PMC7655220

[63] Si ML, Zhu S, Wu H, Lu Z, Wu F, Mo YY. MiR-21-mediated tumor growth. Oncogene. 2007;26(19):2799–803. 17072344 10.1038/sj.onc.1210083

[64] Singh VK, Rajak N, Singh Y, Singh AK, Giri R, Garg N. Role of microrna-21 in prostate cancer progression and metastasis: molecular mechanisms to therapeutic targets. Ann Surg Oncol. 2024;31(7):4795–808. 38758485 10.1245/s10434-024-15453-z

[65] Dan T, Shastri AA, Palagani A, et al. MiR-21 plays a dual role in tumor formation and cytotoxic response in breast tumors. Cancers (Basel). 2021. 10.3390/cancers13040888. 33672628 10.3390/cancers13040888 PMC7924198

[66] Qian S, Liu J, Liao W, Wang F. METTL3 promotes non-small-cell lung cancer growth and metastasis by inhibiting FDX1 through copper death-associated pri-miR-21-5p maturation. Epigenomics. 2023;15(23):1237–55. 38126112 10.2217/epi-2023-0230

[67] Sheedy FJ, Palsson-McDermott E, Hennessy EJ, et al. Negative regulation of TLR4 via targeting of the proinflammatory tumor suppressor PDCD4 by the microRNA miR-21. Nat Immunol. 2010;11(2):141–7. 19946272 10.1038/ni.1828

[68] Xue J, Xiao T, Wei S, et al. Mir-21-regulated M2 polarization of macrophage is involved in arsenicosis-induced hepatic fibrosis through the activation of hepatic stellate cells. J Cell Physiol. 2021;236(8):6025–41. 33481270 10.1002/jcp.30288

[69] Das A, Ganesh K, Khanna S, Sen CK, Roy S. Engulfment of apoptotic cells by macrophages: a role of microRNA-21 in the resolution of wound inflammation. J Immunol. 2014;192(3):1120–9. 24391209 10.4049/jimmunol.1300613 PMC4358325

[70] Yang J, Shuai J, Siow L, et al. Microrna-146a-loaded magnesium silicate nanospheres promote bone regeneration in an inflammatory microenvironment. Bone Res. 2024;12(1):2. 38221522 10.1038/s41413-023-00299-0 PMC10788347

[71] Monteiro CGJ, Vieira EM, Emerick C, et al. Ozonated oil effect for prevention of medication-related osteonecrosis of the jaw (MRONJ) in rats undergoing zoledronic acid therapy. Clin Oral Invest. 2021;25(12):6653–9. 10.1007/s00784-021-03951-3 33895916

[72] Ning H, Wu X, Wu Q, et al. Microfiber-reinforced composite hydrogels loaded with rat adipose-derived stem cells and BMP-2 for the treatment of medication-related osteonecrosis of the jaw in a rat model. ACS Biomater Sci Eng. 2019;5(5):2430–43. 33405751 10.1021/acsbiomaterials.8b01468

[73] Suyama K, Otsuru M, Nakamura N, et al. Bone resection methods in medication-related osteonecrosis of the jaw in the mandible: an investigation of 206 patients undergoing surgical treatment. J Dent Sci. 2024;19(3):1758–69. 39035329 10.1016/j.jds.2023.10.007 PMC11259631

[74] Soutome S, Otsuru M, Hayashida S, et al. Periosteal reaction of medication-related osteonecrosis of the jaw (MRONJ): clinical significance and changes during conservative therapy. Support Care Cancer. 2021;29(11):6361–8. 33884506 10.1007/s00520-021-06214-9

[75] Kuang Y, Zhai J, Xiao Q, Zhao S, Li C. Polysaccharide/mesoporous silica nanoparticle-based drug delivery systems: a review. Int J Biol Macromol. 2021;193(Pt A):457–73. 34710474 10.1016/j.ijbiomac.2021.10.142

[76] She X, Chen L, Yi Z, et al. Tailored mesoporous silica nanoparticles for controlled drug delivery: platform fabrication, targeted delivery, and computational design and analysis. Mini Rev Med Chem. 2018;18(11):976–89. 27145854 10.2174/1389557516666160505114814

[77] Wei W, Ma Y, Yao X, et al. Advanced hydrogels for the repair of cartilage defects and regeneration. Bioact Mater. 2021;6(4):998–1011. 33102942 10.1016/j.bioactmat.2020.09.030 PMC7557878

[78] Tang F, Li L, Chen D. Mesoporous silica nanoparticles: synthesis, biocompatibility and drug delivery. Adv Mater (Deerfield Beach, Fla). 2012;24(12):1504–34. 10.1002/adma.201104763 22378538

[79] Christoforidou T, Giasafaki D, Andriotis EG, et al. Oral drug delivery systems based on ordered mesoporous silica nanoparticles for modulating the release of aprepitant. Int J Mol Sci. 2021. 10.3390/ijms22041896. 33672949 10.3390/ijms22041896 PMC7917702

[80] Park-Min KH, Mun SH, Bockman R, McDonald MM. New horizons: translational aspects of osteomorphs. J Clin Endocrinol Metab. 2024;109(5):e1373–8. 38060842 10.1210/clinem/dgad711 PMC11031245

