# Supplementary material for: Localized therapeutic strategy based on microRNA-21-loaded mesoporous silica nanoparticles hydrogel improves bone repair in medication-related osteonecrosis of the jaw
Source: J Orthop Surg Res. 2025 Dec 23;20:1079. doi: 10.1186/s13018-025-06503-7 (PMC12723889; doi:10.1186/s13018-025-06503-7)
Supplement: Supplementary file 1 [file 13018_2025_6503_MOESM1_ESM.docx]

**Supplementary Figure 1. Effects of MSN alone on osteoclast differentiation and NF-κB/PDCD4 signaling in RAW264.7 cells.** (A) RT-qPCR analysis of osteoclast-related genes (Rela, Nfatc1, Acp5, Ctsk) and Pdcd4 and miR-21 expression in RAW264.7 cells treated with MSN+miR-21, miR-21, or blank control. (B) Western blot analysis showing the expression of p-p65, p65, CTSK, and PDCD4 in each group. GAPDH was used as a loading control. (C) Quantification of TRAP-positive multinucleated cells per field. (D) Representative images of TRAP staining showing osteoclast differentiation under different treatment conditions. Scale bar = 500 μm.

**Supplementary Figure 2. In situ hybridization (RISH) analysis of miR-21 expression in mandibular bone sections from different treatment groups.** Representative low-magnification (15×) and high-magnification (250×) images show the localization of miR-21-positive signals (brown) within the defect area.

**Supplementary Figure 3. Histological evaluation of femoral bone structure after different treatments.** Representative HE-stained sections (15× magnification) of femoral tissues from Control, Blank, MSN, and MSN+miR-21 groups show intact cortical and trabecular bone structures across all groups. The MSN+miR-21 group displayed dense and continuous trabecular organization comparable to the Control group, indicating no structural abnormalities or adverse bone effects after local treatment. Scale bar = 5000 μm.
