# Supplementary figures and images for: Localized therapeutic strategy based on microRNA-21-loaded mesoporous silica nanoparticles hydrogel improves bone repair in medication-related osteonecrosis of the jaw
Source: J Orthop Surg Res. 2025 Dec 23;20:1079. doi: 10.1186/s13018-025-06503-7 (PMC12723889; doi:10.1186/s13018-025-06503-7)

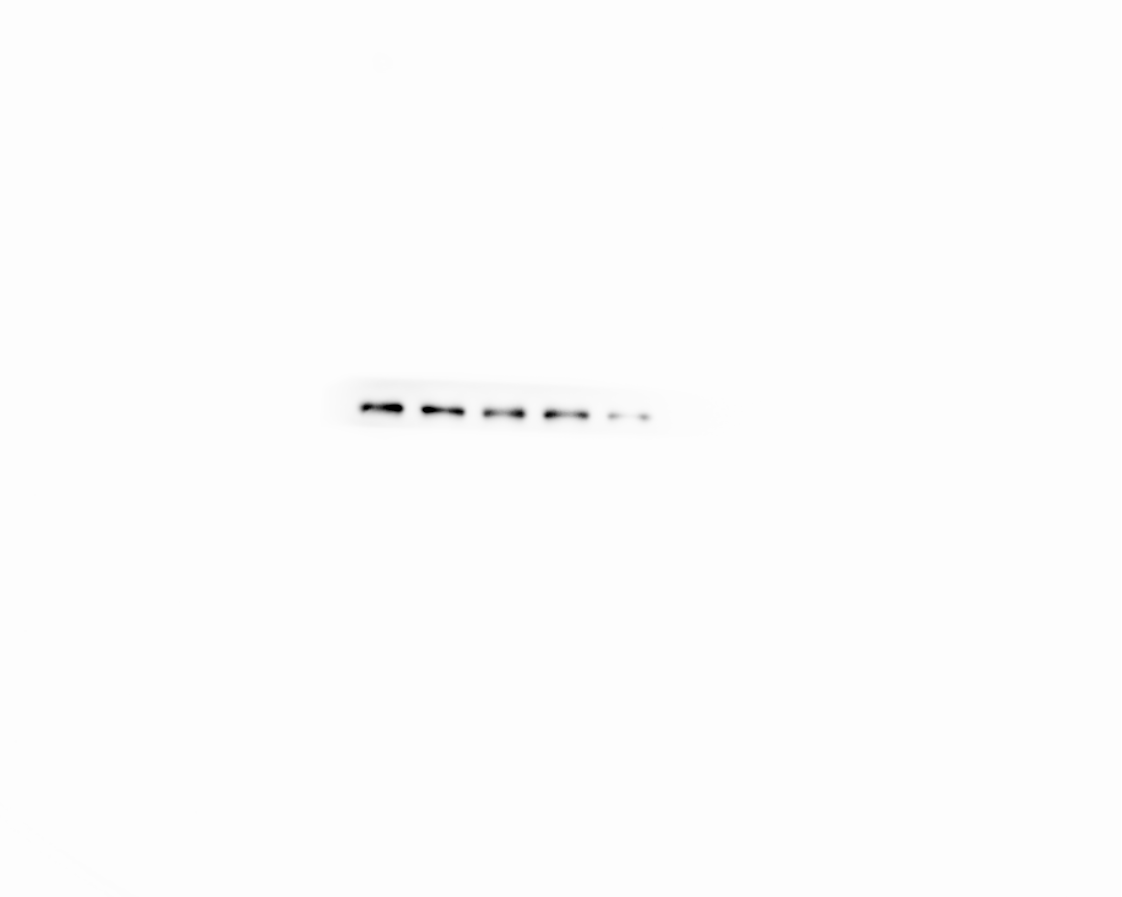

Supplement: Supplementary file 2 [file 13018_2025_6503_MOESM2_ESM.zip › Original protein bands/Figure 1E. CTSK.tif]

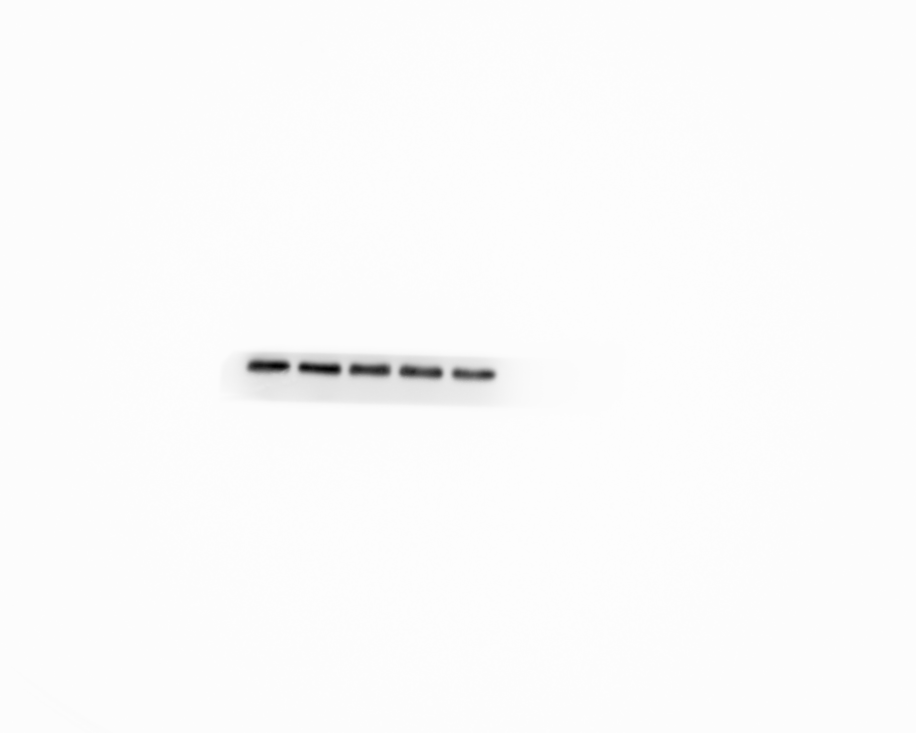

Supplement: Supplementary file 2 [file 13018_2025_6503_MOESM2_ESM.zip › Original protein bands/Figure 1E. GAPDH.tif]

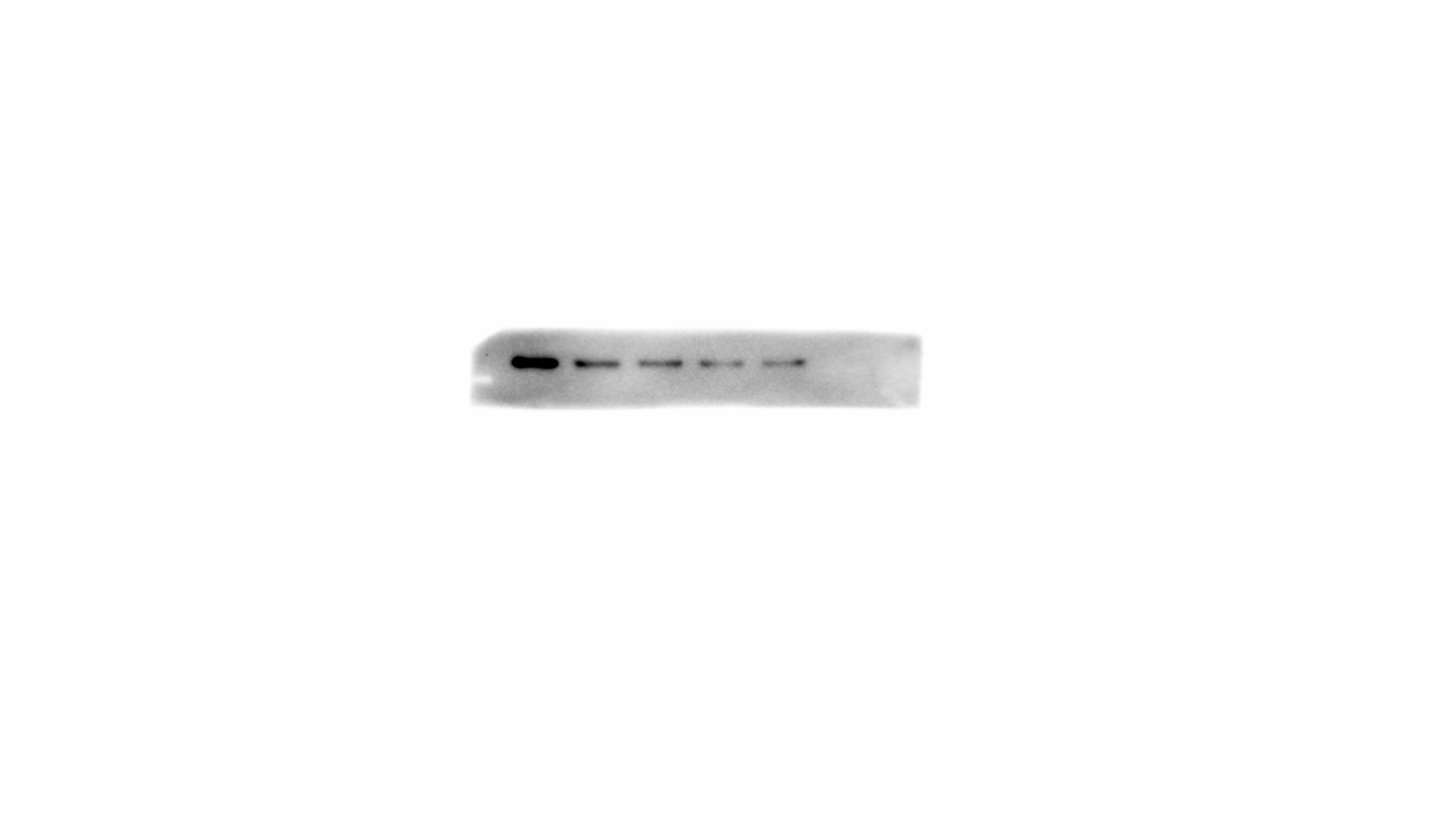

Supplement: Supplementary file 2 [file 13018_2025_6503_MOESM2_ESM.zip › Original protein bands/Figure 1E. p-p65.tif]

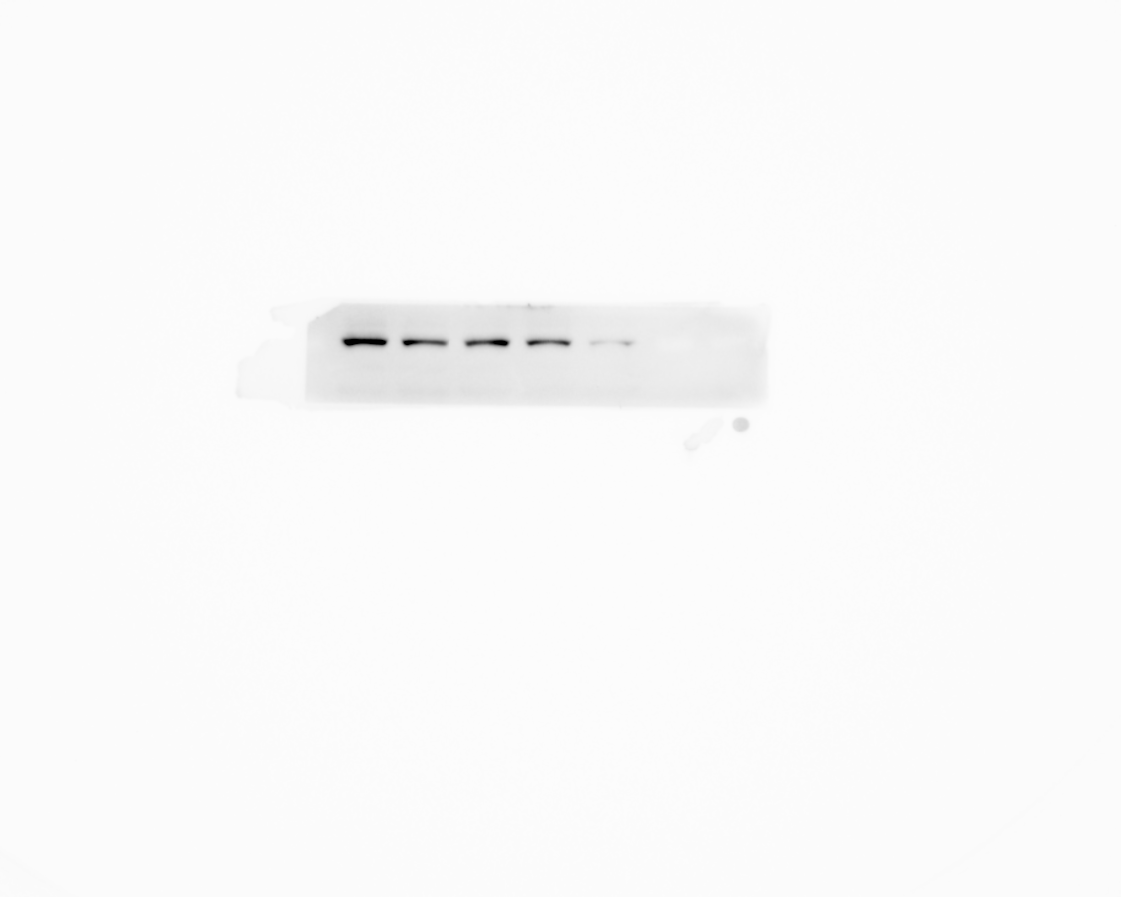

Supplement: Supplementary file 2 [file 13018_2025_6503_MOESM2_ESM.zip › Original protein bands/Figure 1E. p65.tif]

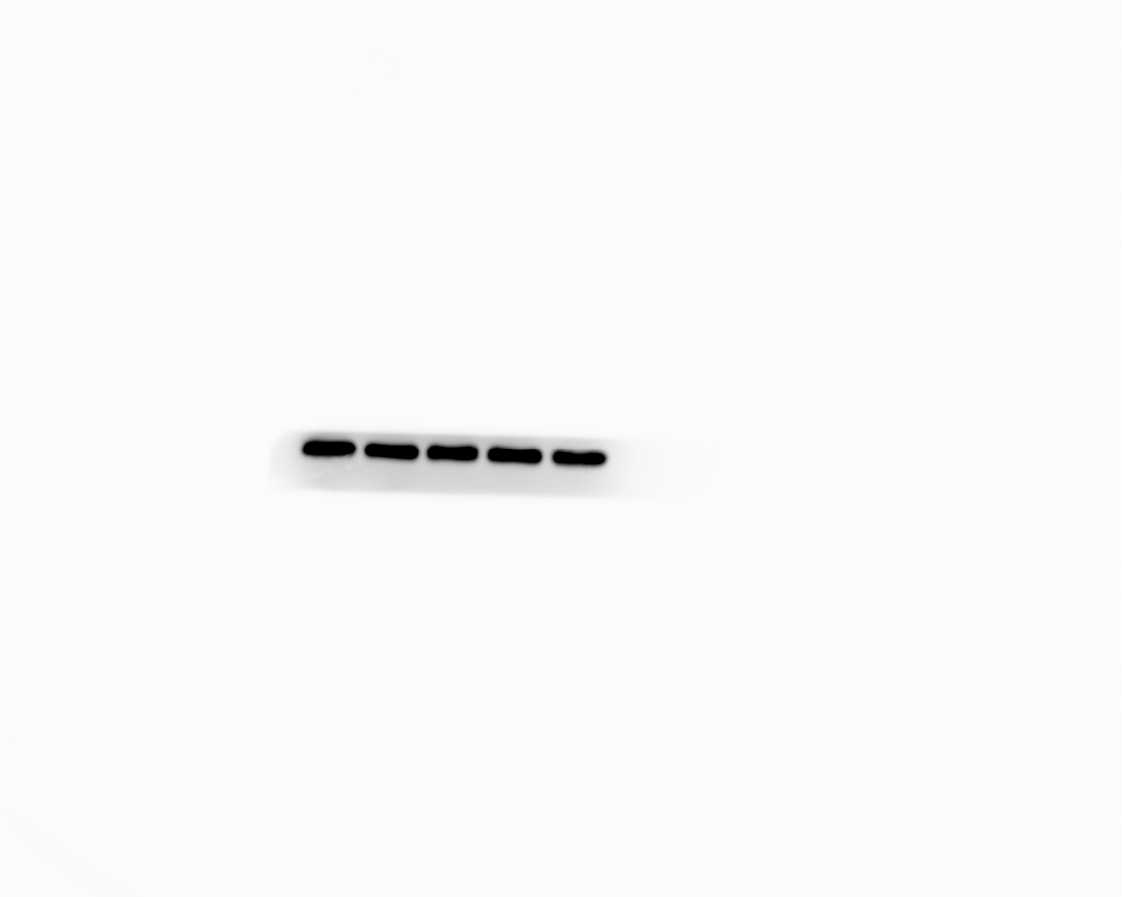

Supplement: Supplementary file 2 [file 13018_2025_6503_MOESM2_ESM.zip › Original protein bands/Figure 1H. GAPDH.tif]

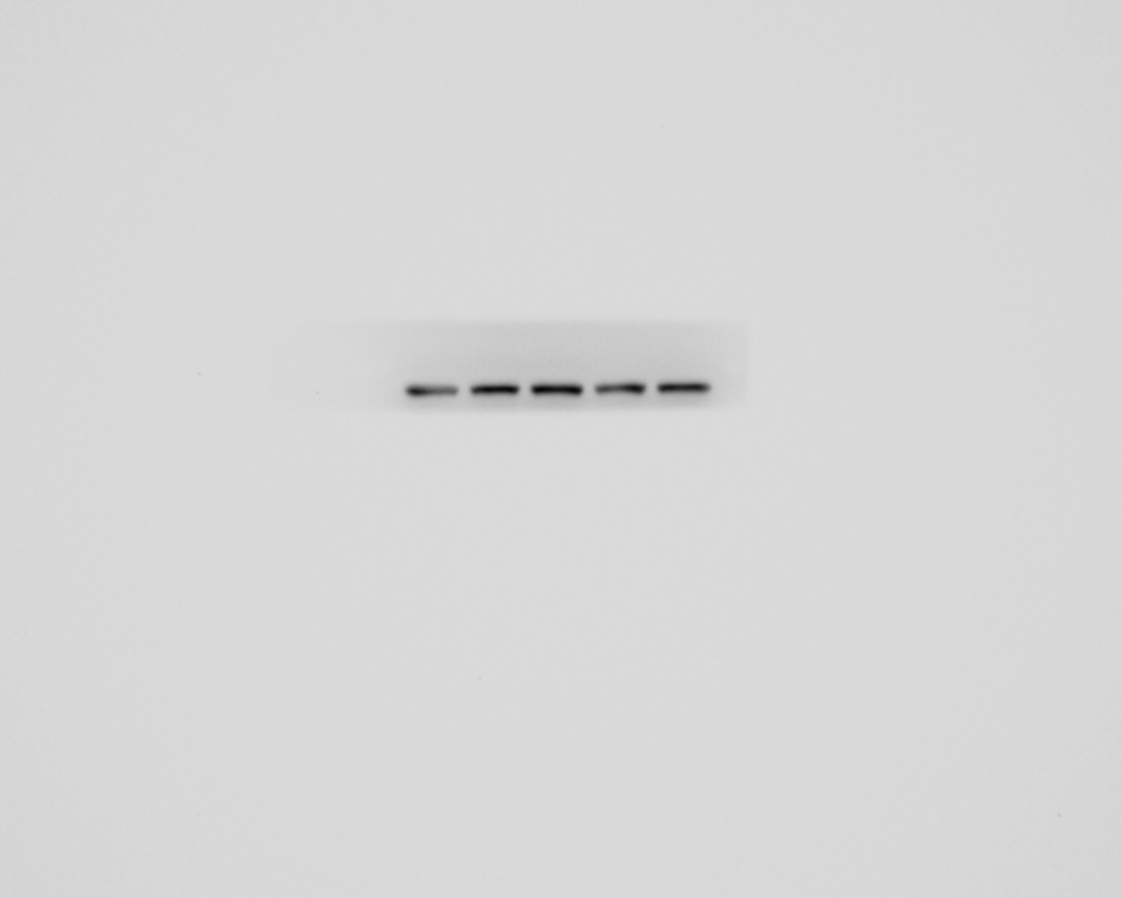

Supplement: Supplementary file 2 [file 13018_2025_6503_MOESM2_ESM.zip › Original protein bands/Figure 1H. PDCD4.tif]

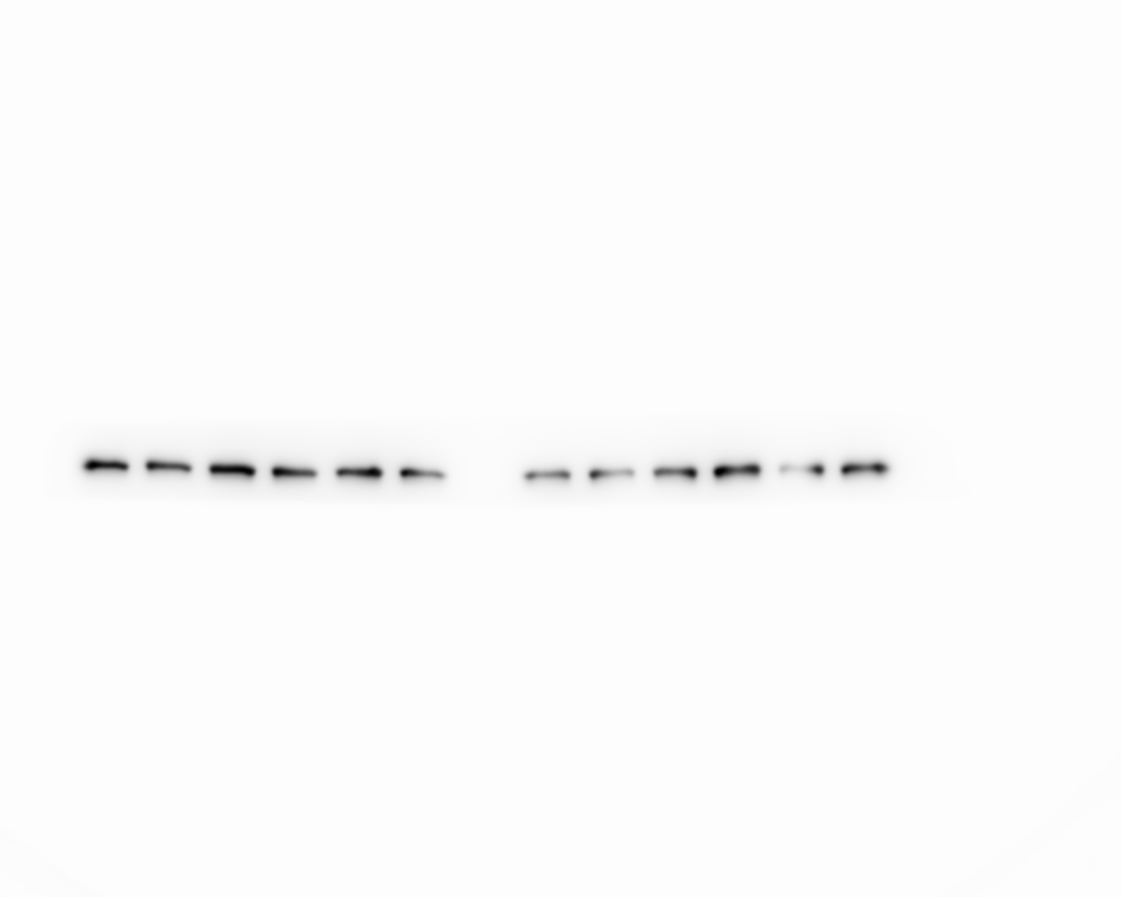

Supplement: Supplementary file 2 [file 13018_2025_6503_MOESM2_ESM.zip › Original protein bands/Figure 2D. CTSK.tif]

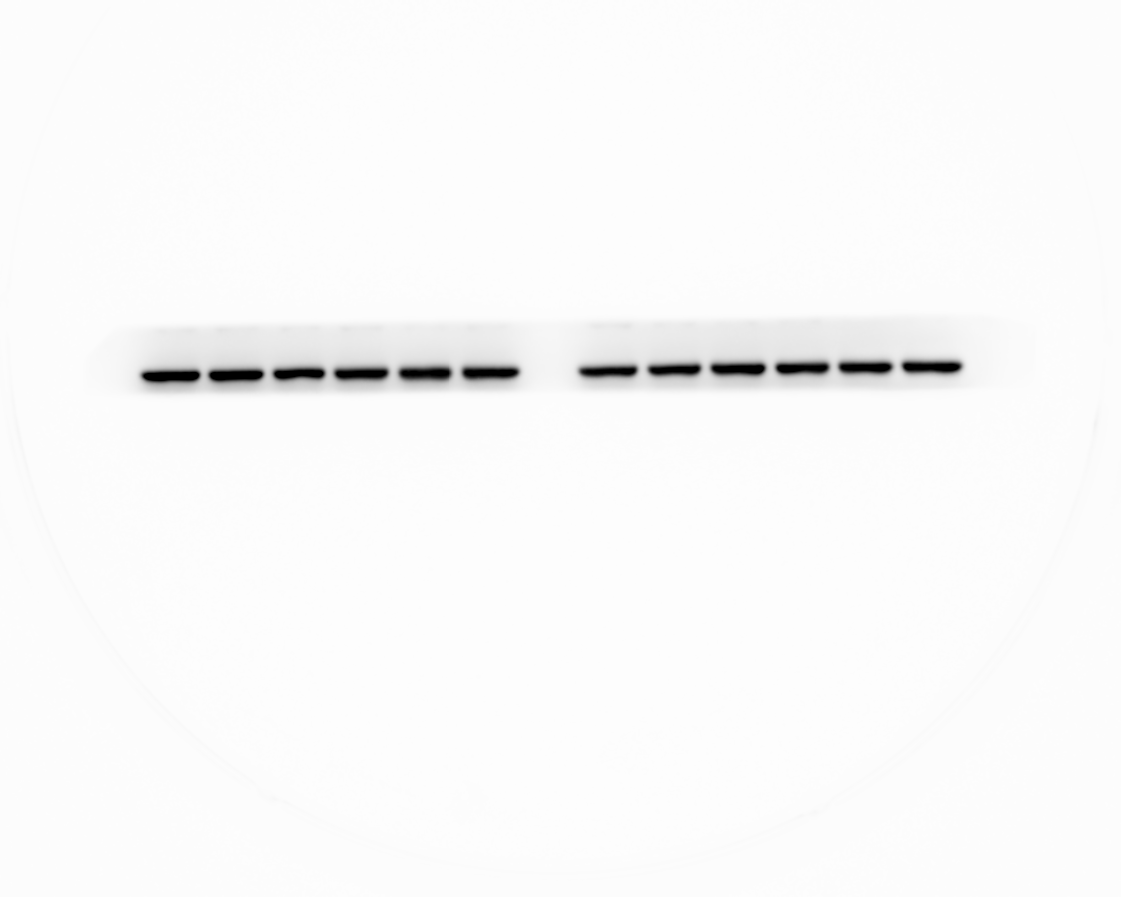

Supplement: Supplementary file 2 [file 13018_2025_6503_MOESM2_ESM.zip › Original protein bands/Figure 2D. GAPDH.tif]

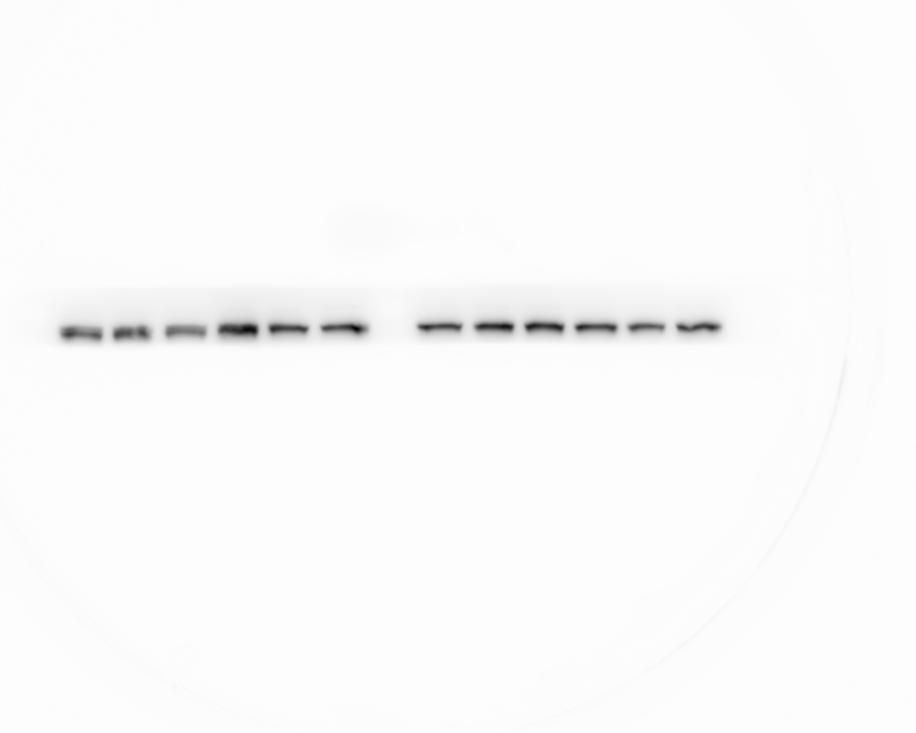

Supplement: Supplementary file 2 [file 13018_2025_6503_MOESM2_ESM.zip › Original protein bands/Figure 2D. PDCD4.tif]

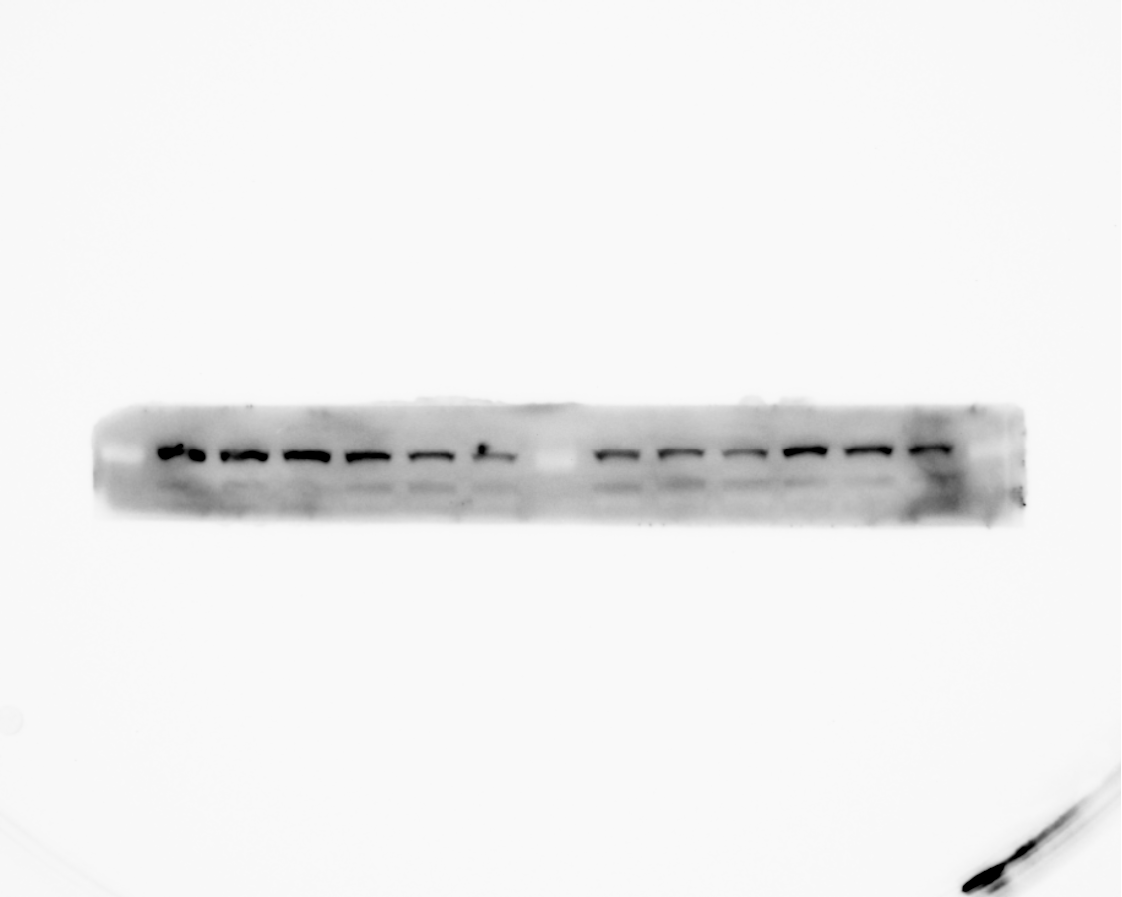

Supplement: Supplementary file 2 [file 13018_2025_6503_MOESM2_ESM.zip › Original protein bands/Figure 2D. p-p65.tif]

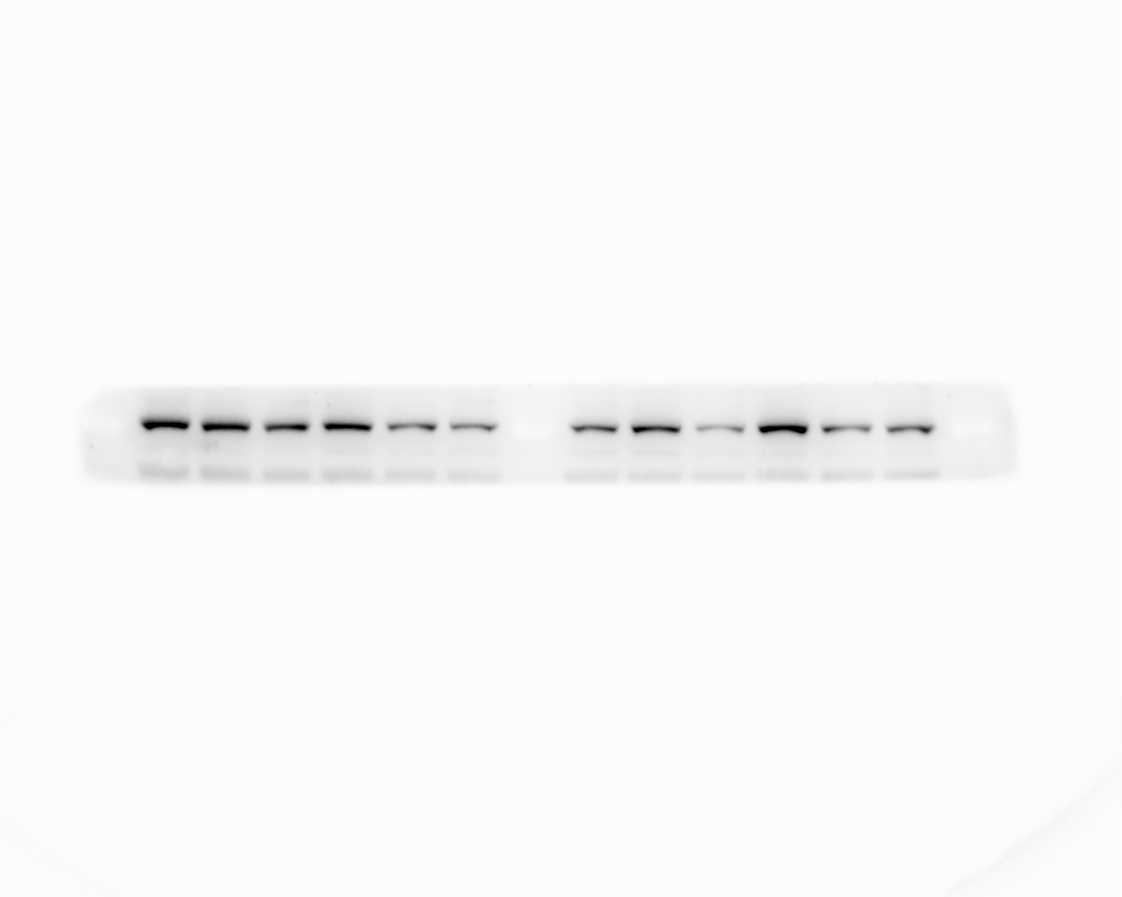

Supplement: Supplementary file 2 [file 13018_2025_6503_MOESM2_ESM.zip › Original protein bands/Figure 2D. p65.tif]

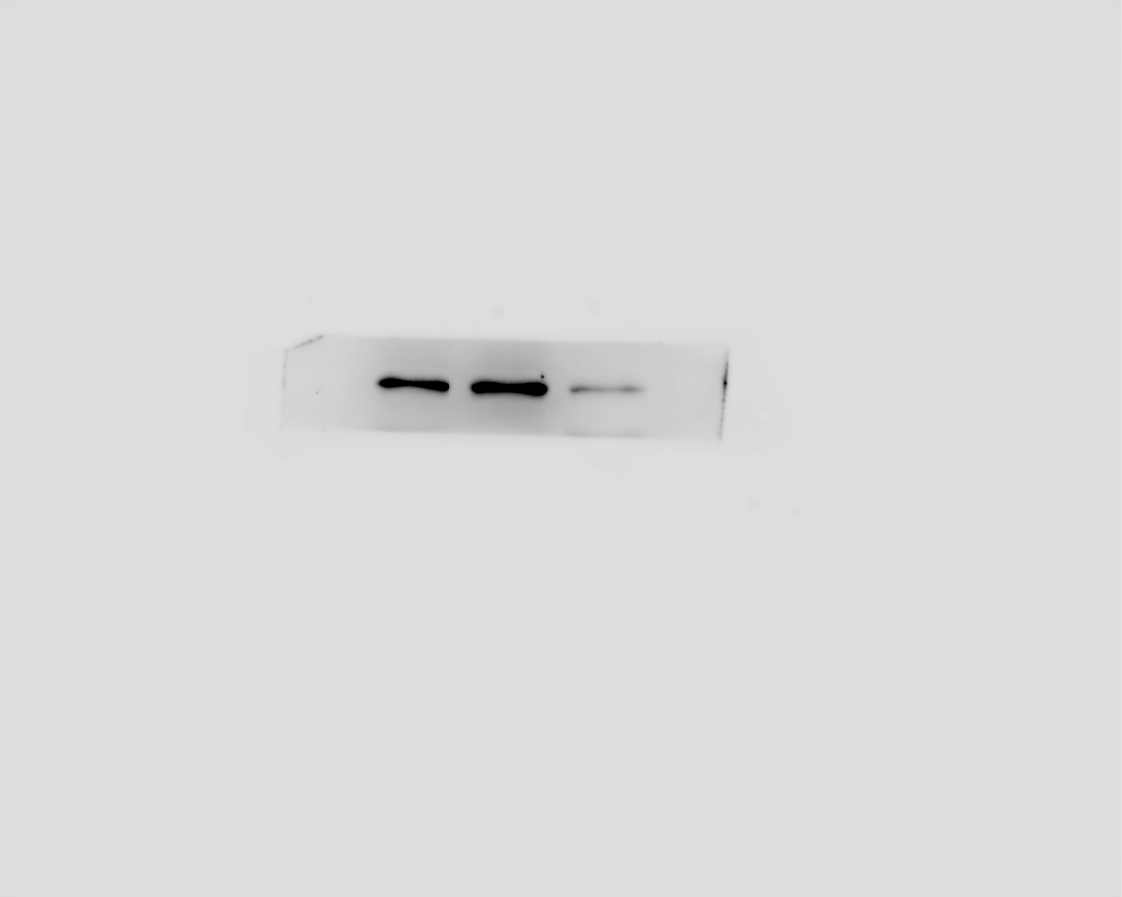

Supplement: Supplementary file 2 [file 13018_2025_6503_MOESM2_ESM.zip › Original protein bands/Supplementary Figure 1B. CTSK.tif]

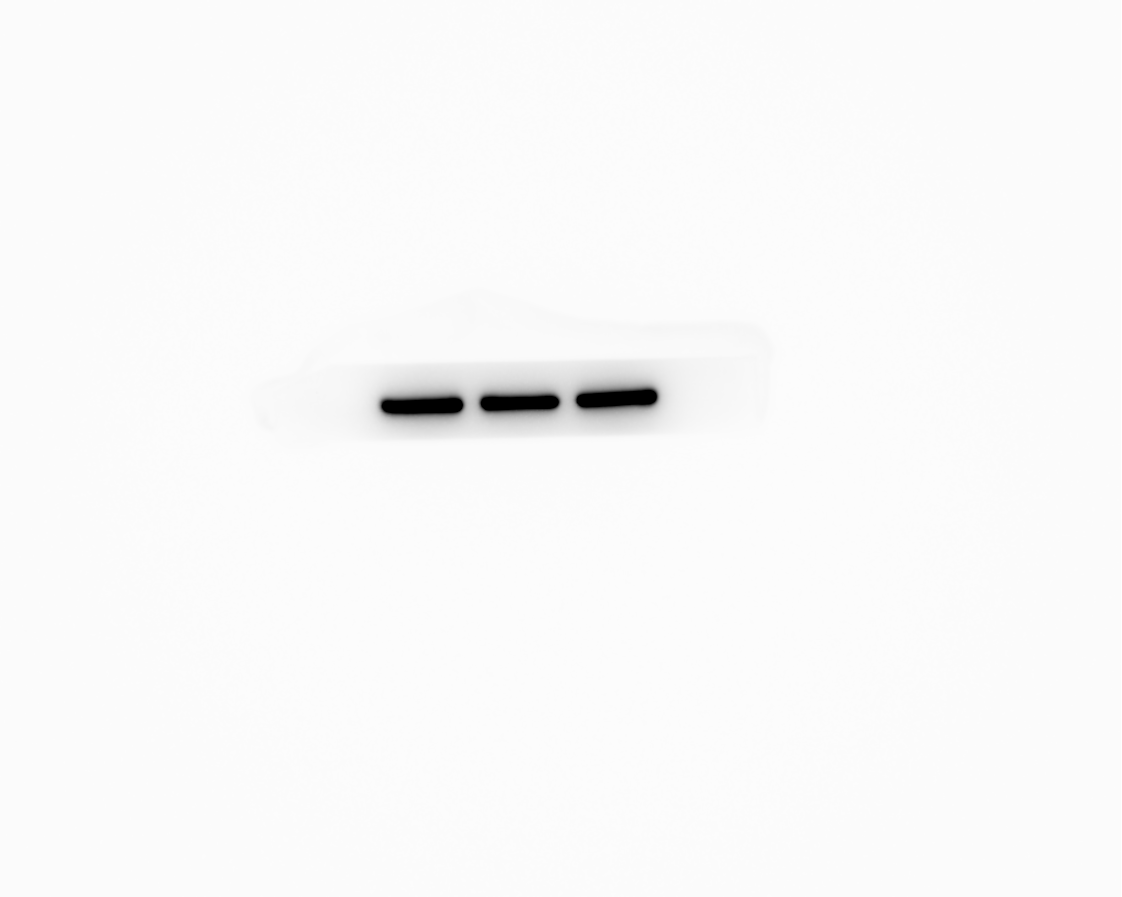

Supplement: Supplementary file 2 [file 13018_2025_6503_MOESM2_ESM.zip › Original protein bands/Supplementary Figure 1B. GAPDH.tif]

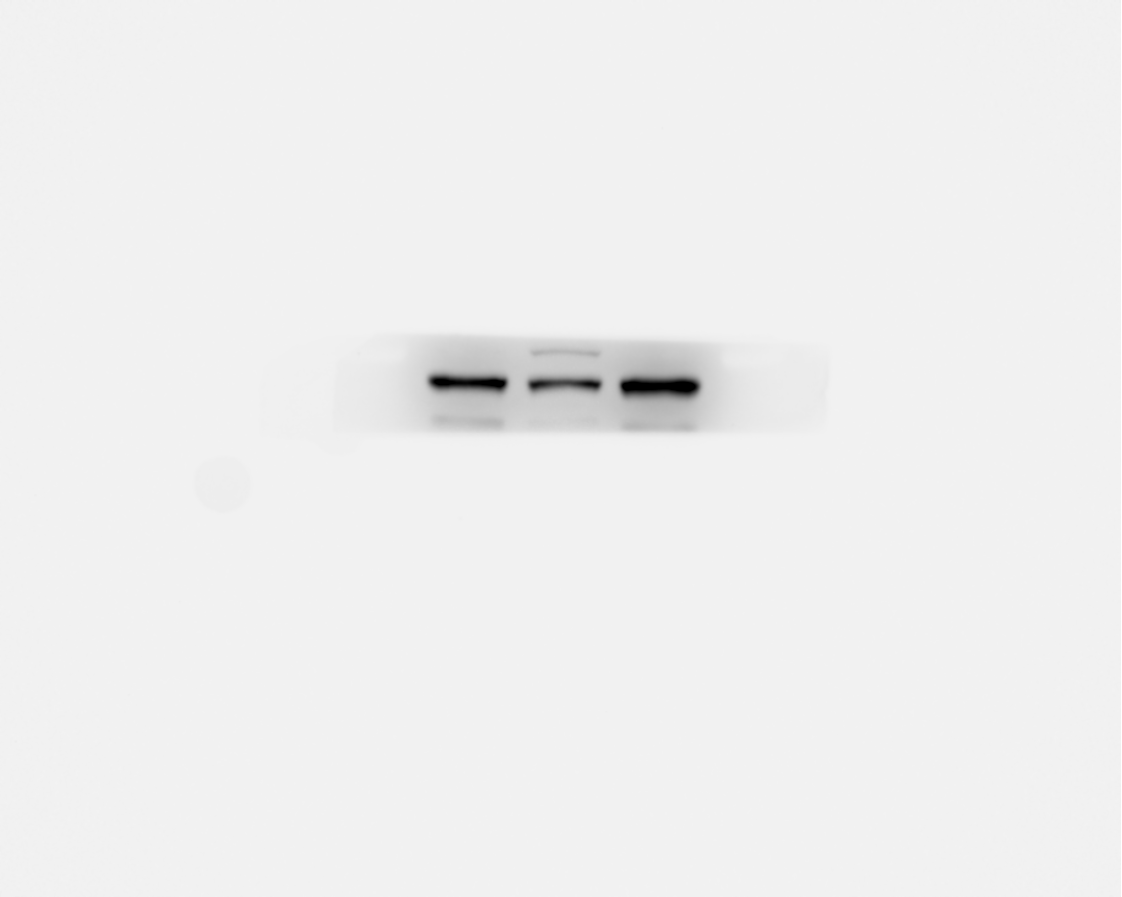

Supplement: Supplementary file 2 [file 13018_2025_6503_MOESM2_ESM.zip › Original protein bands/Supplementary Figure 1B. PDCD4.tif]

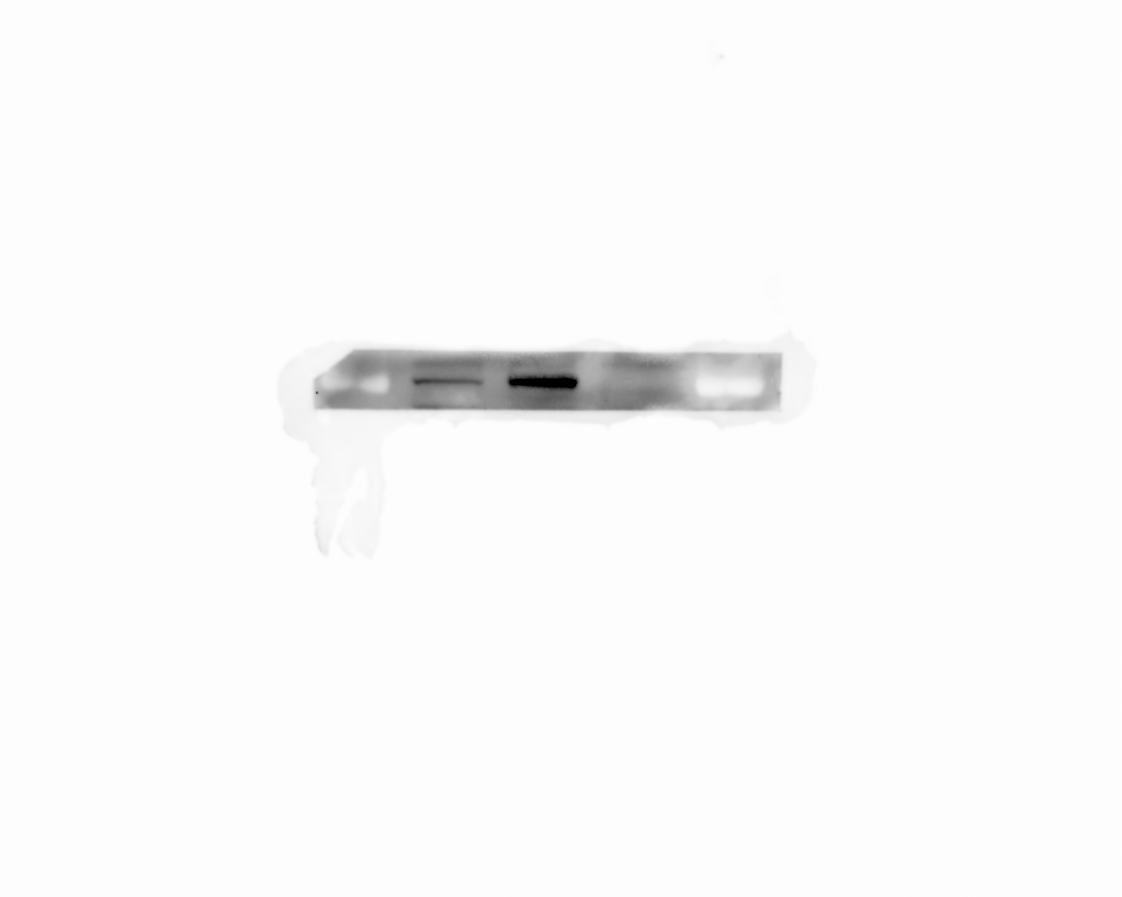

Supplement: Supplementary file 2 [file 13018_2025_6503_MOESM2_ESM.zip › Original protein bands/Supplementary Figure 1B. p-p65.tif]

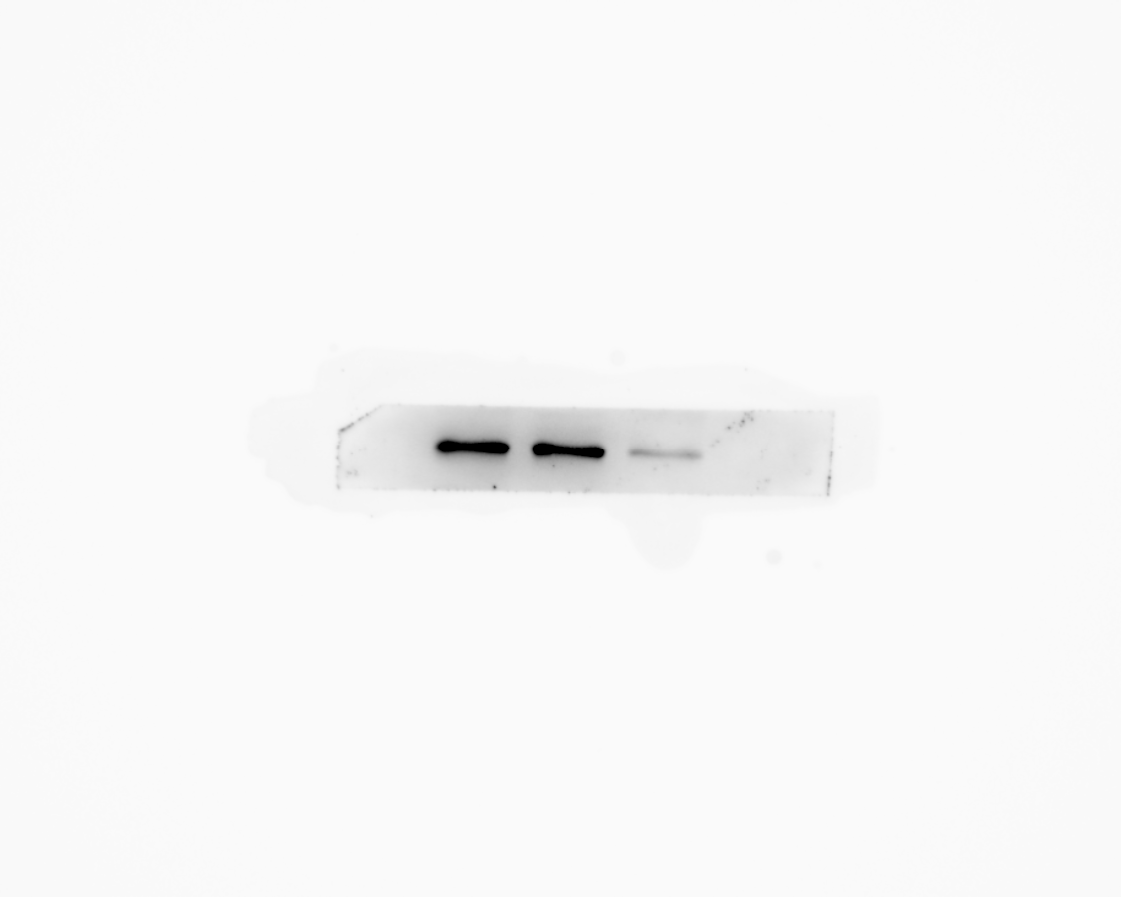

Supplement: Supplementary file 2 [file 13018_2025_6503_MOESM2_ESM.zip › Original protein bands/Supplementary Figure 1B. p65.tif]

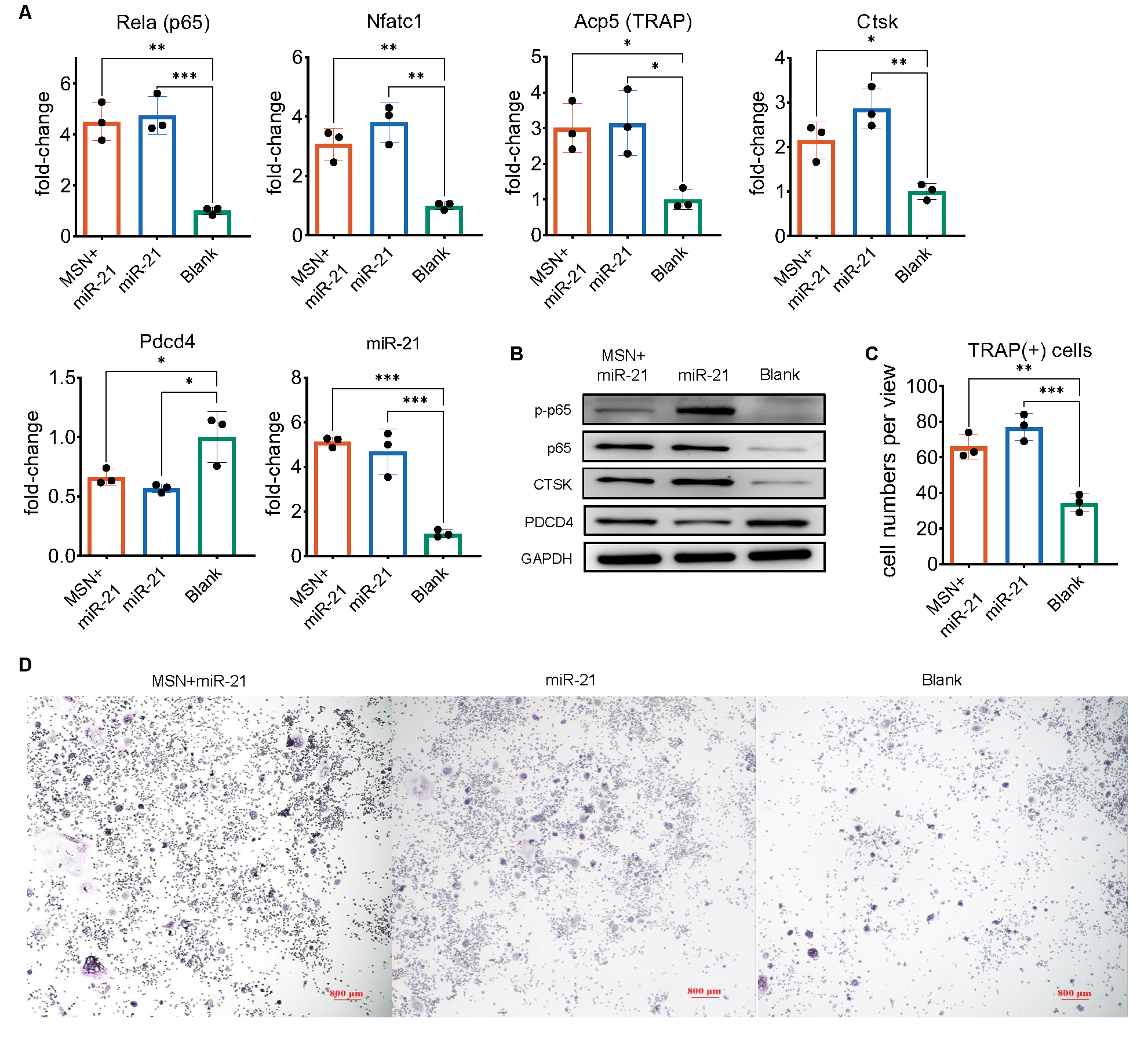

Supplement: Supplementary file 3 [file 13018_2025_6503_MOESM3_ESM.tiff]

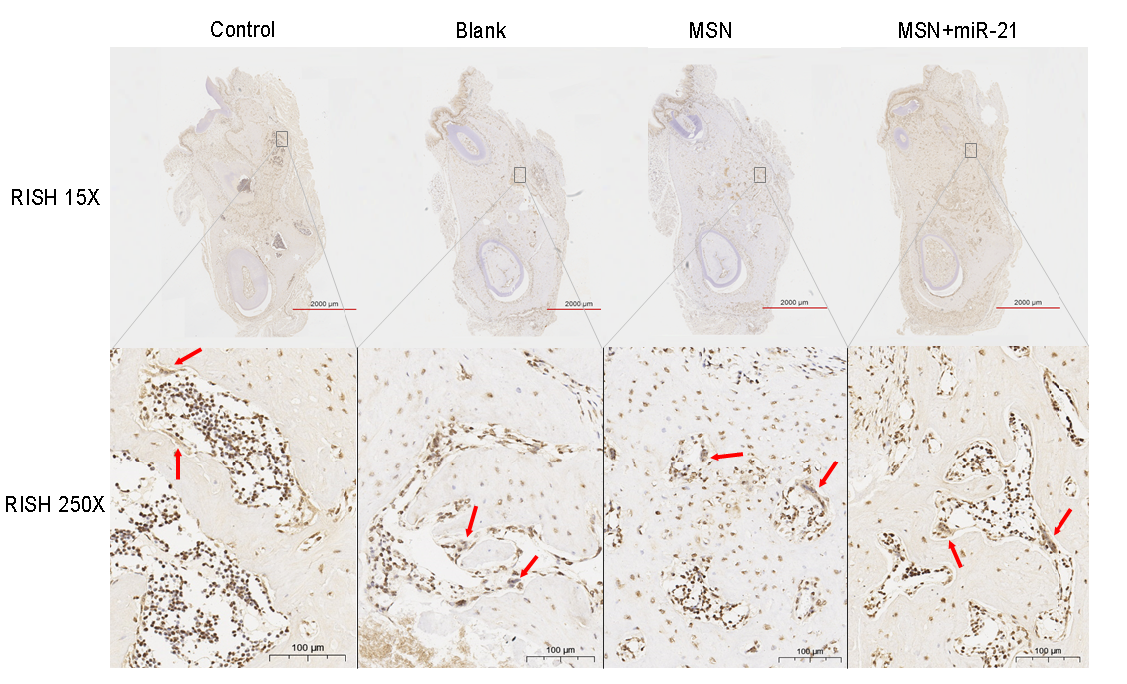

Supplement: Supplementary file 4 [file 13018_2025_6503_MOESM4_ESM.tiff]

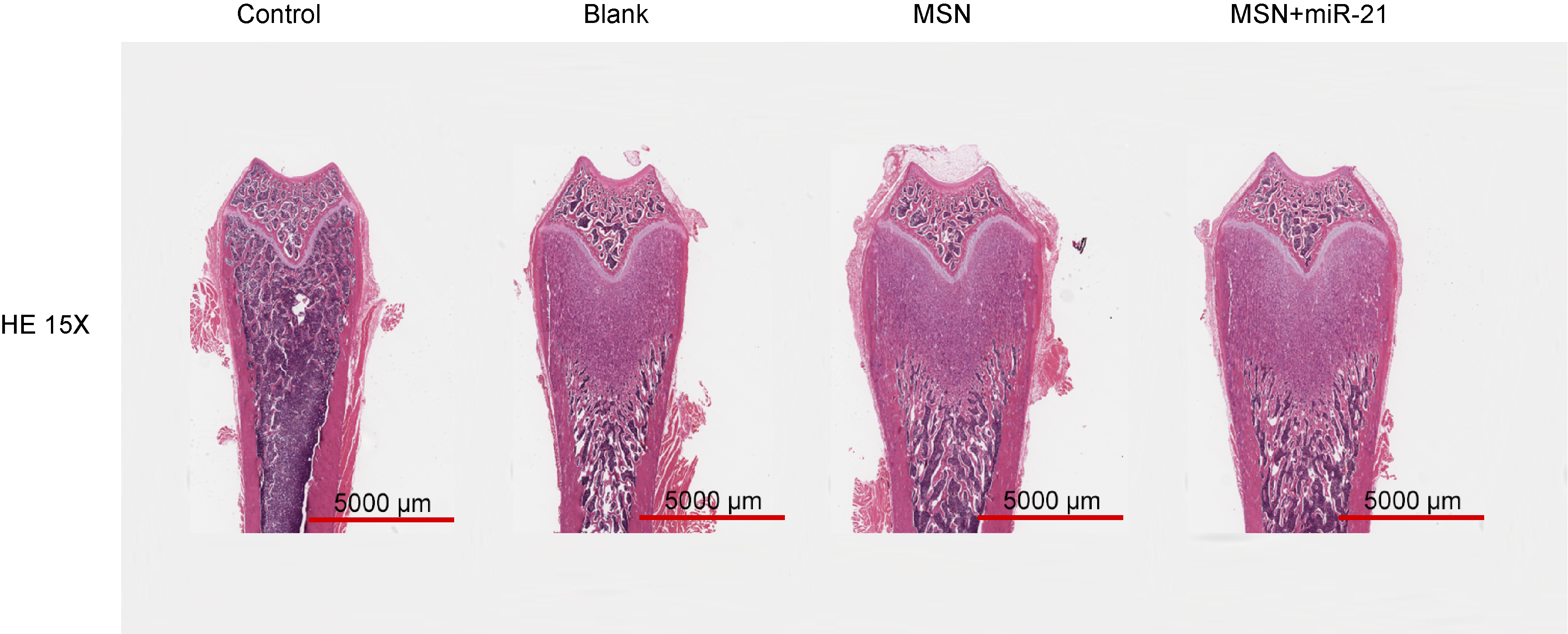

Supplement: Supplementary file 5 [file 13018_2025_6503_MOESM5_ESM.tif]
